# Supplementary material for: Ambient hydrogenation of solid aromatics enabled by a high entropy alloy nanocatalyst
Source: Nat Commun. 2024 Jul 10;15:5806. doi: 10.1038/s41467-024-50009-5 (PMC11236972; doi:10.1038/s41467-024-50009-5)
Supplement: Supplementary file 1 — Supplymentary Information [file 41467_2024_50009_MOESM1_ESM.pdf]

Supplementary Information

Ambient hydrogenation of solid aromatics enabled by a high entropy alloy nanocatalyst

Zekun Jing [1, #], Yakun Guo [1, #], Qi Wang [1, #], Xinrong Yan [2], Guozong Yue [3], Zhendong Li [1], Hanwen Liu [4], Ruixuan Qin [5], Changyin Zhong [3], Mingzhen Li [1, 6], Dingguo Xu [2, ✉], Yunxi Yao [3, ✉], Yonggang Yao, [4, ✉], and Maobing Shuai [3, ✉]

[1] Science and Technology on Surface Physics and Chemistry Laboratory, Jiangyou 621908, China

[2] College of Chemistry, Sichuan University, Chengdu 610065, China

[3] Institute of Materials, China Academy of Engineering Physics, Mianyang 621907, China

[4] State Key Laboratory of Materials Processing and Die & Mould Technology, School of Materials Science and Engineering, Huazhong University of Science and Technology, Wuhan 430074, China

[5] Department of Chemistry, College of Chemistry and Chemical Engineering, Xiamen University, Xiamen 361005, China

[6] State Key Laboratory of Environment-friendly Energy Materials, Southwest University of Science and Technology, Mianyang 621010, China

[#] These authors contributed equally.

✉ email: shuaimb@sina.com; yaoyg@hust.edu.cn; yaoyunxi@caep.cn; dgxu@scu.edu.cn.

Supplementary Figures

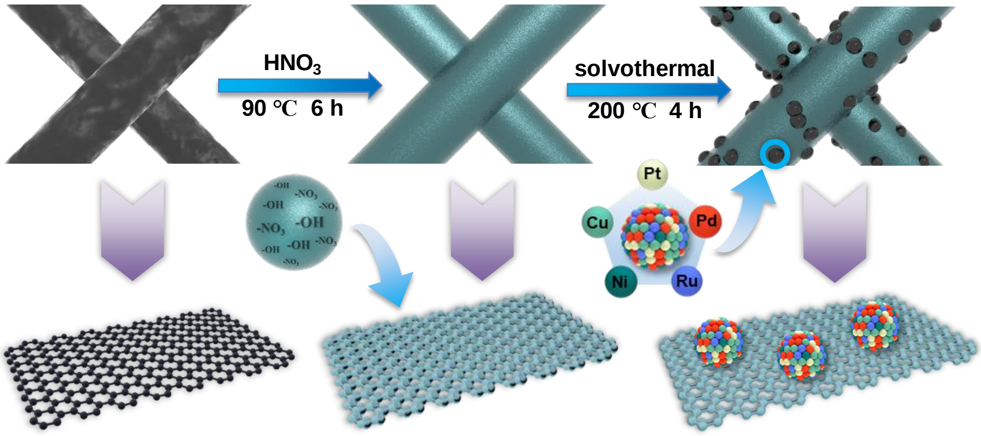

Supplementary Fig. 1 The preparation schematic graph of PdPtRuCuNi/CNFs high-entropy alloy nanocatalyst.

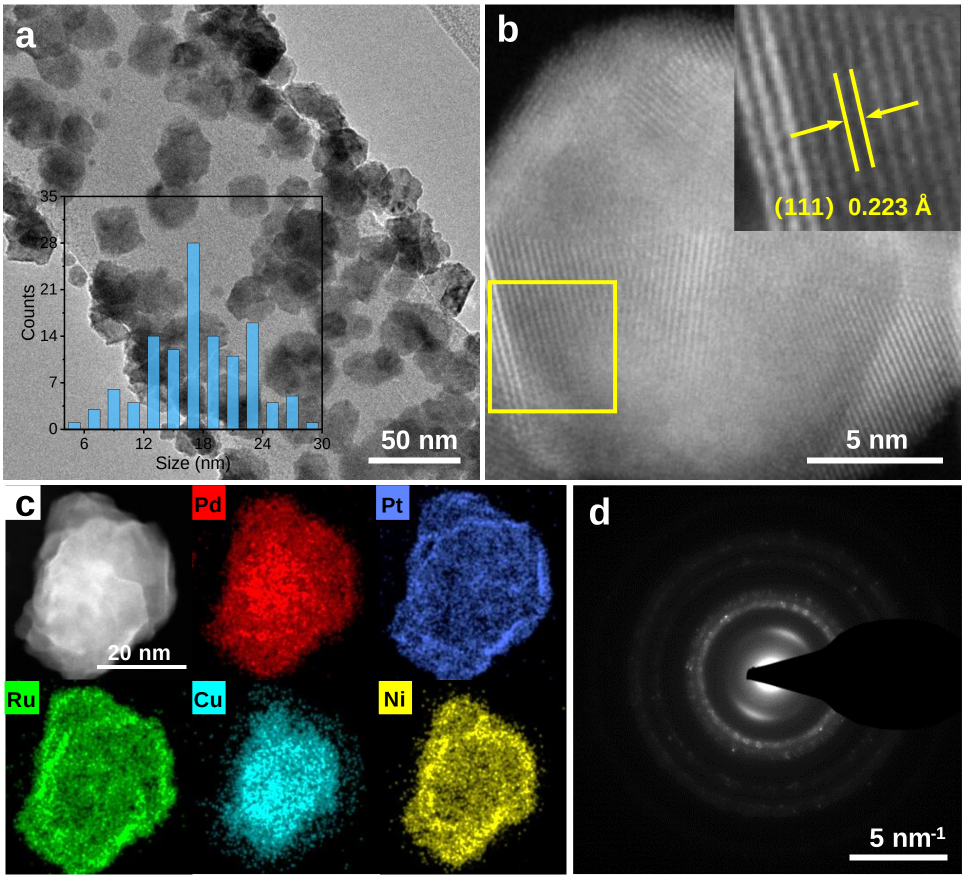

Supplementary Fig. 2 The TEM characteristic of PdPtRuCuNi/CNFs. a TEM image and the distribution of nanoparticles size. **b** HRTEM image. **c** EDS mapping. **d** Selected area electron diffraction (SAED) image. The EDS mapping indicates that the Pd, Pt, Ru, Cu, and Ni elements distribute uniformly on the nanoparticles.

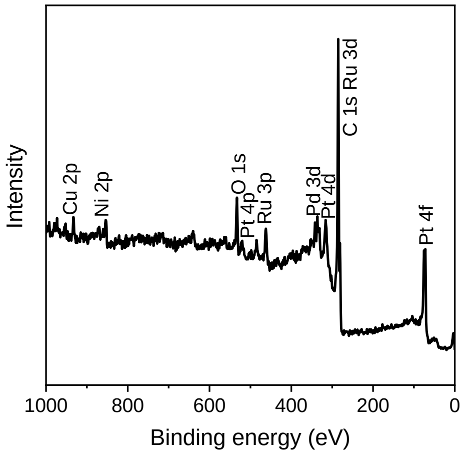

Supplementary Figure. 3 XPS full spectra of PdPtRuCuNi/CNFs.

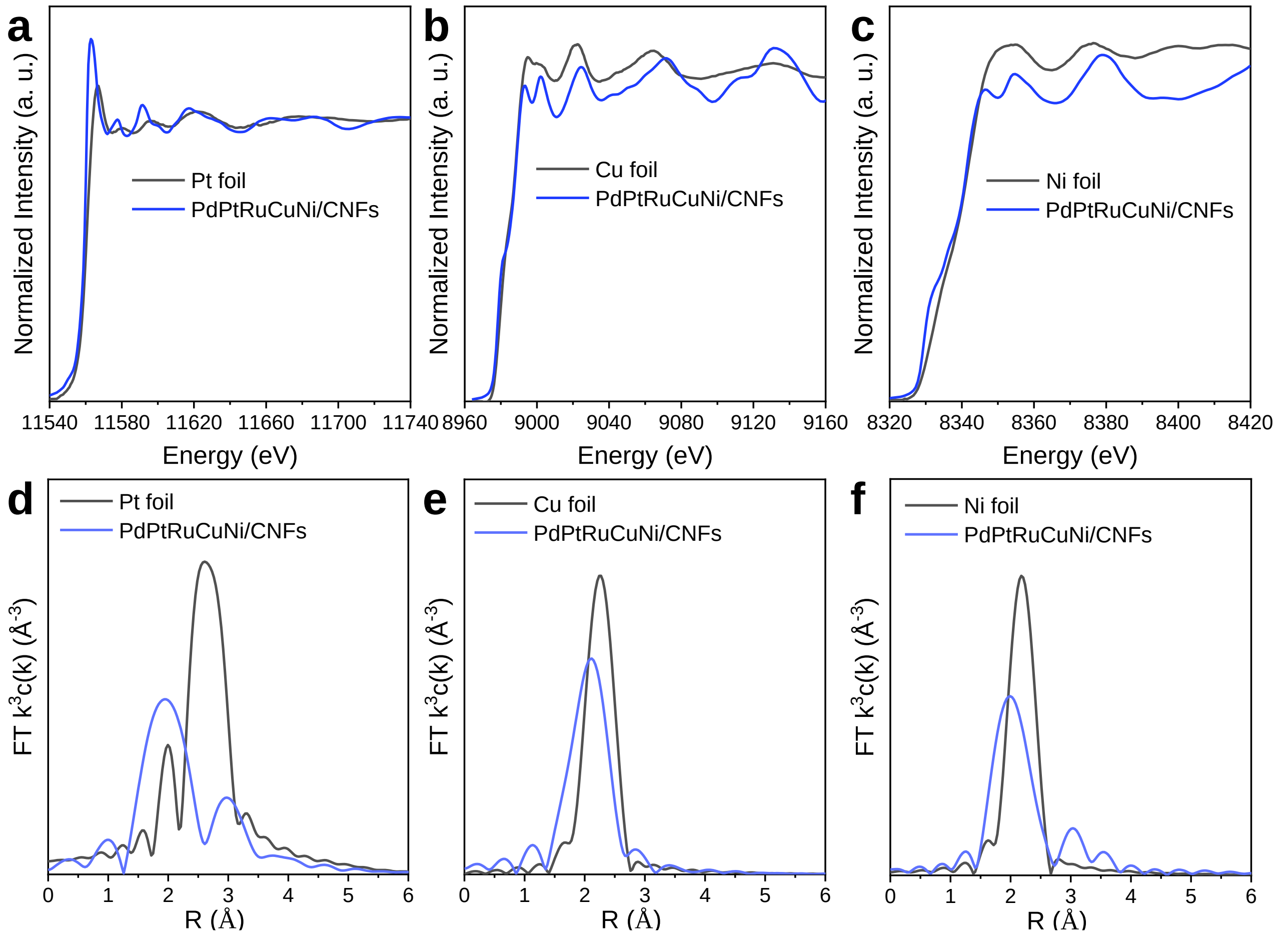

Supplementary Fig. 4 The characterization of X-ray near-side absorption. a-c The X-ray absorption near edge structure (XANES) spectra of PdPtRuCuNi/CNFs and monometal. **a** The Pt L3-edge. **b** The Cu K-edge. **c** The Ni K-edge. **d-f** Fourier transform EXAFS spectra of PdPtRuCuNi/CNFs and monometal. **d** Pt. **e** Cu. **f** Ni.

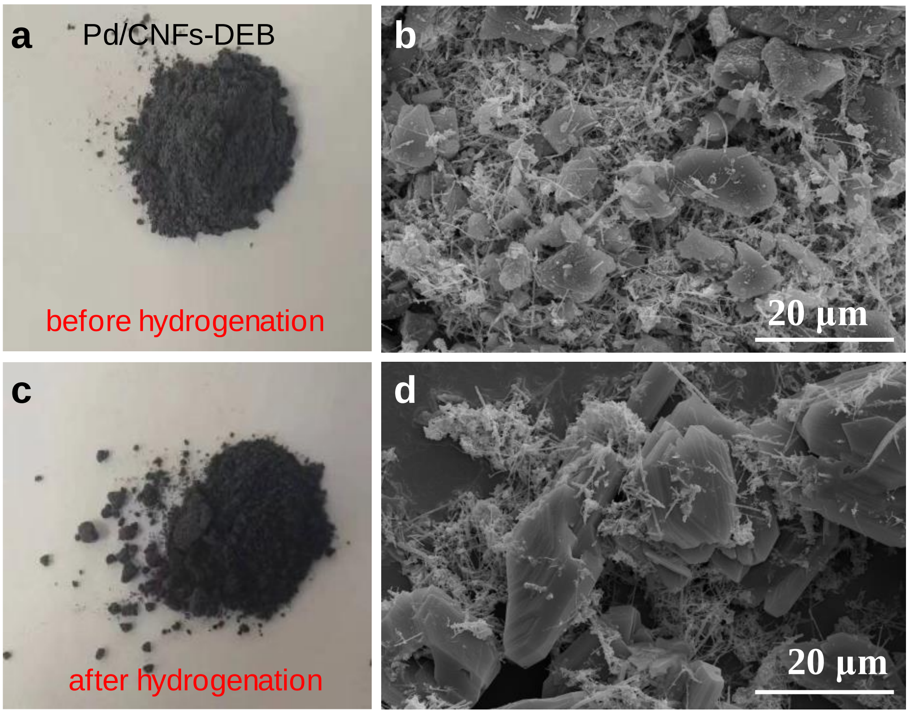

Supplementary Fig. 5 The morphology of Pd/CNFs-DEB composites before and after hydrogenation. **a**, **c** The digital images. **b**, **d** SEM images.

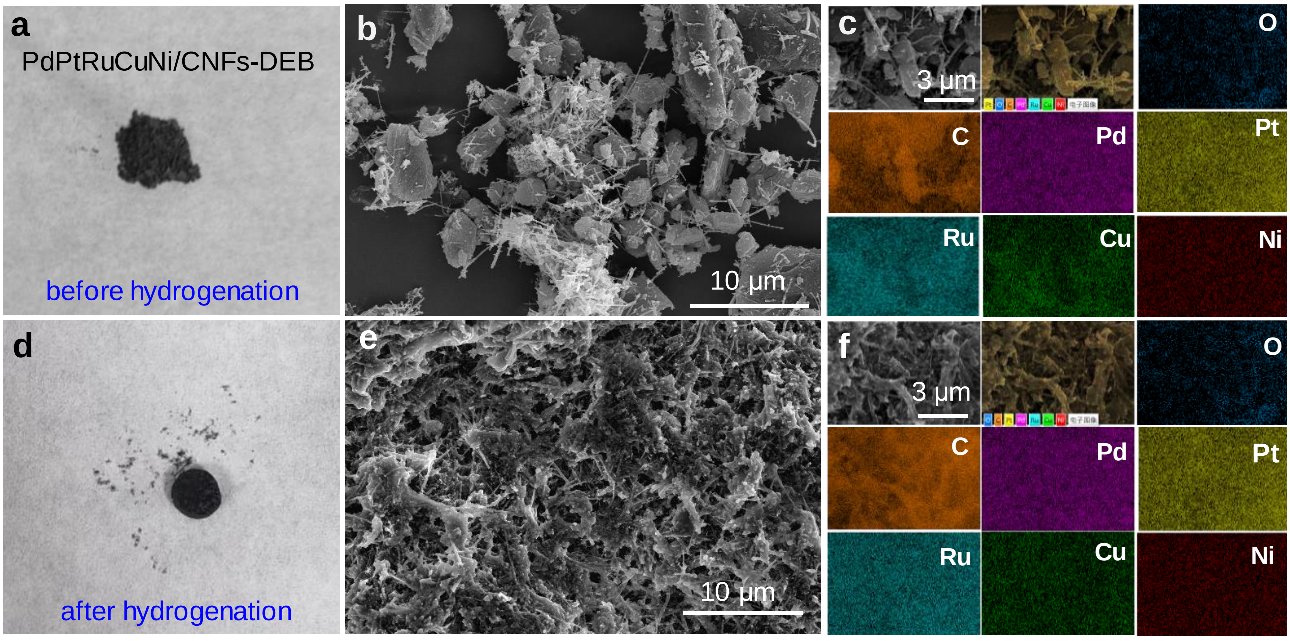

Supplementary Fig. 6 The morphology and element distribution of PdPtRuCuNi/CNFs-DEB composites before and after hydrogenation. **a**, **d** The digital images. **b**, **e** SEM images. **c**, **f** Element mapping of Pd, Pt, Ru, Cu, Ni, C and O. The Pd/CNFs-DEB composite (Supplementary Figure 5) is the loose powder before and after hydrogenation, while the PdPtRuCuNi/CNFs-DEB changes from loose powder to melt after hydrogenation.

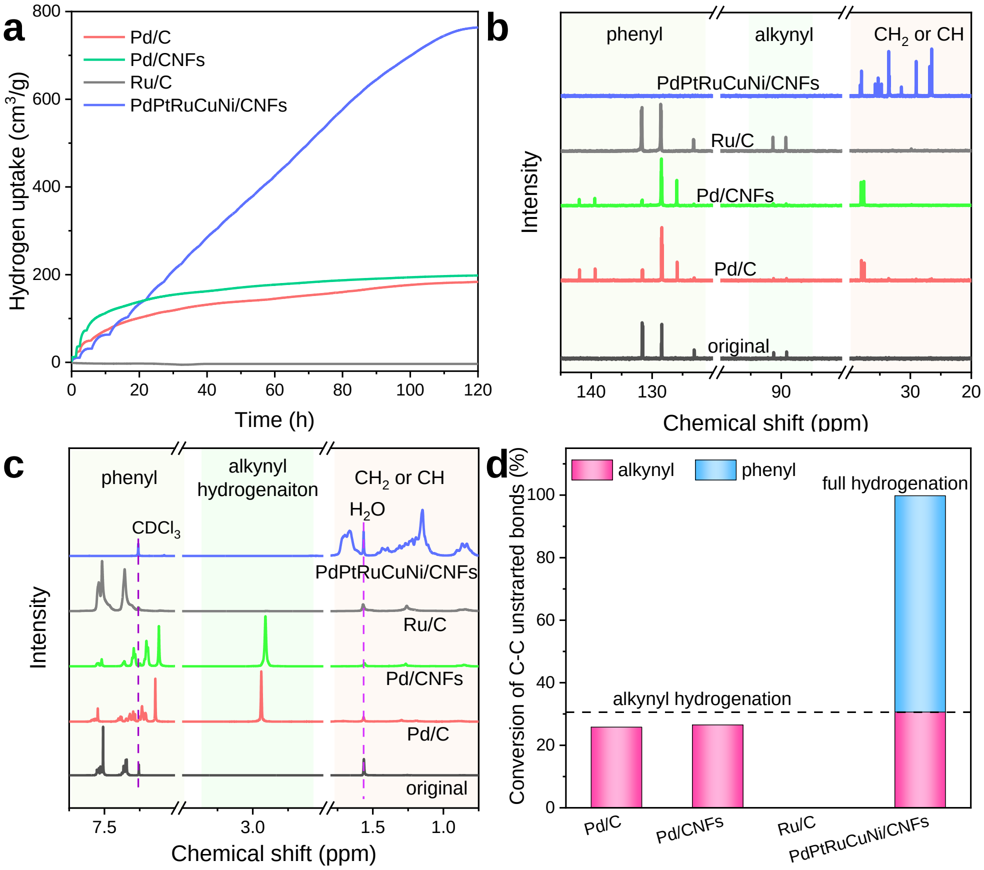

Supplementary Fig. 7 The catalytic hydrogenation by different catalysts including PdPtRuCuNi/CNFs, Pd/CNFs, the commercial Pd/C, and Pt/C. **a** The hydrogen uptake curves with time. **b** ^{13}C and **c** ^{1}H NMR of DEB before and after hydrogenation. **d** The conversion of C-C unsaturated bonds (alkynyl and phenyl groups) in the hydrogenated DEB molecules.

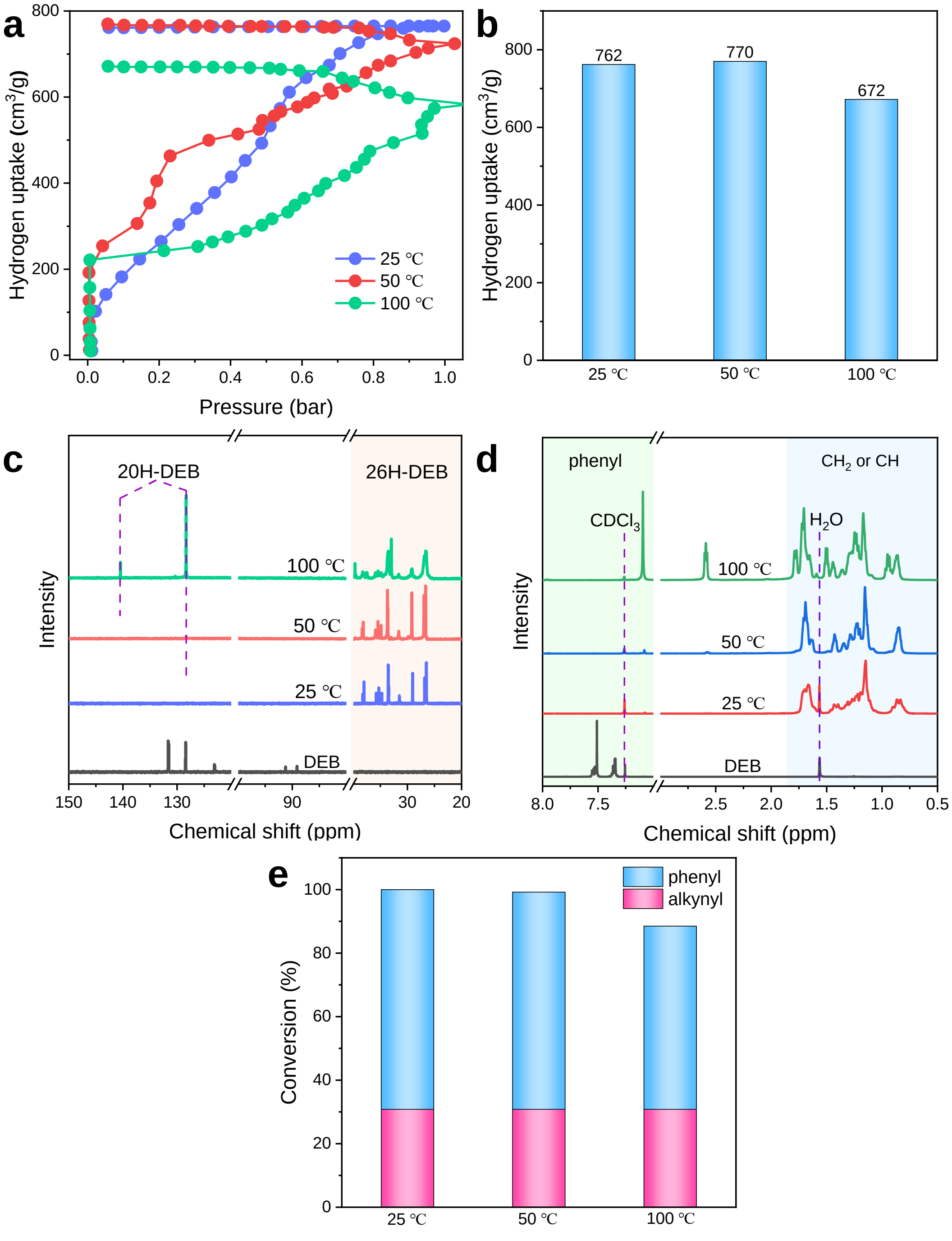

Supplementary Fig. 8 The hydrogenation of PdPtRuCuNi/CNFs-DEB at different temperatures. a Absorption and desorption curves with pressure (absorption: 0-1 bar, desorption 1-0 bar). **b** The final amount of hydrogen uptake. **c** ^{13}C and **d** ^{1}H NMR of DEB before and after hydrogenation. **e** The conversion of C-C unsaturated bonds (alkynyl and phenyl groups) in the hydrogenated DEB molecules.

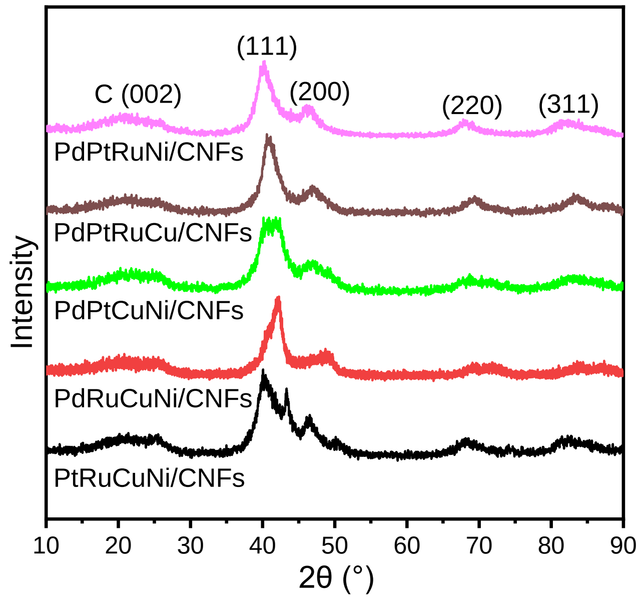

Supplementary Fig. 9 The XRD patterns of quaternary alloy catalysts. The (111) surface of PtRuCuNi has two significant peaks, which means Pd is the crucial factor in forming single-phase alloys.

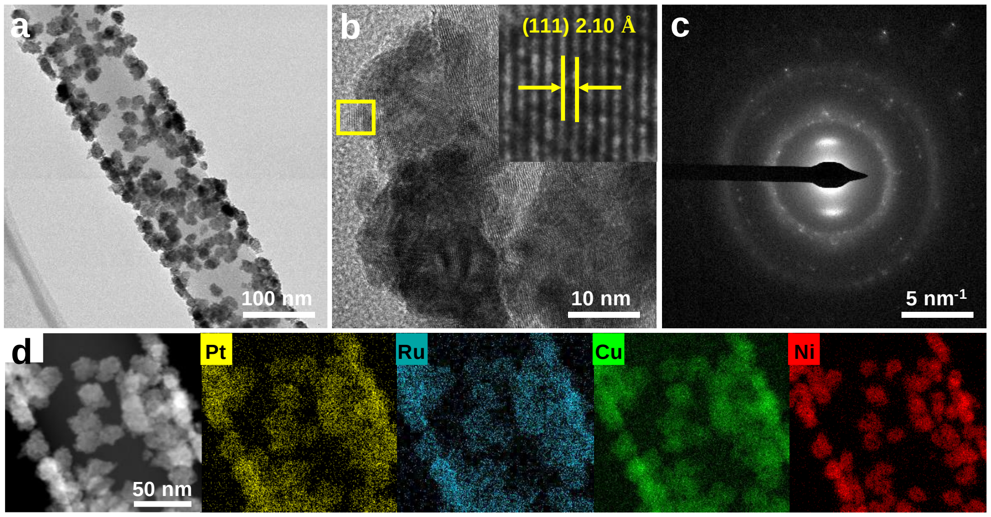

Supplementary Fig. 10 The TEM characterization of PtRuCuNi/CNFs. **a** TEM morphology. **b** HRTEM image. **c** Selective electron diffraction pattern. **d** EDS mapping. The EDS mapping indicates that the Pt, Ru, Cu, and Ni elements distribute uniformly on the nanoparticles.

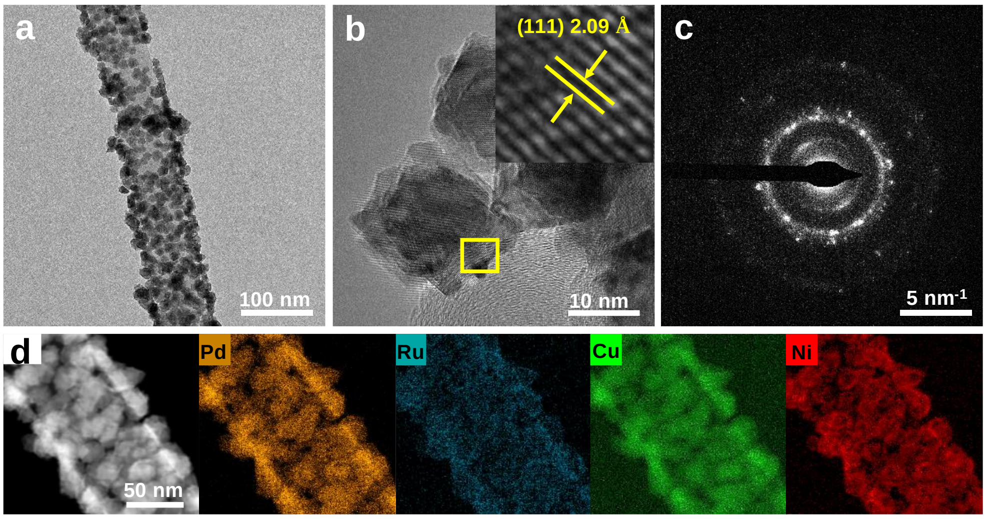

Supplementary Fig.11 The TEM characterization of PdRuCuNi/CNFs. **a** TEM morphology. **b** HRTEM image. **c** Selective electron diffraction pattern. **d** EDS mapping. The EDS mapping indicates that the Pd, Ru, Cu, and Ni elements distribute uniformly on the nanoparticles.

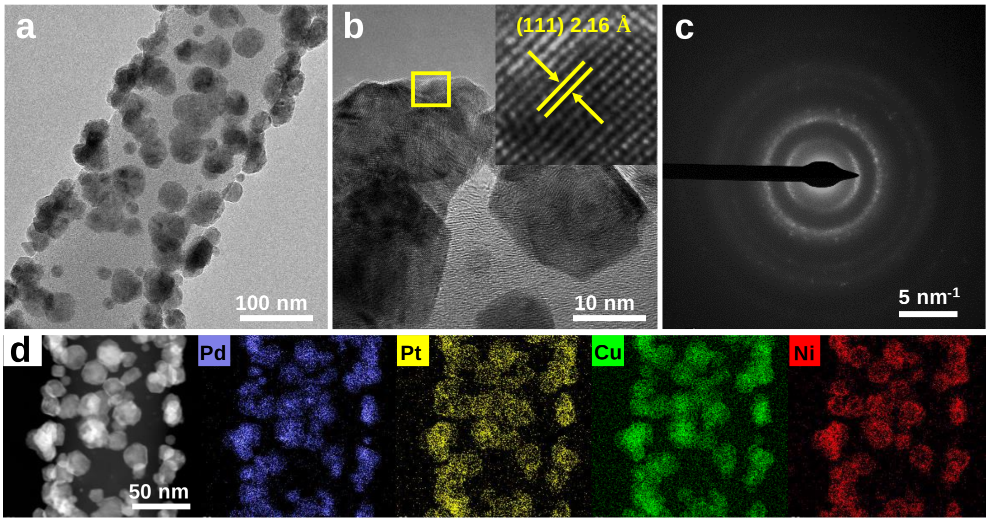

Supplementary Fig. 12 The TEM characterization of PdPtCuNi/CNFs. **a** TEM morphology. **b** HRTEM image. **c** Selective electron diffraction pattern. **d** EDS mapping. The EDS mapping indicates that the Pd, Pt, Cu, and Ni elements distribute uniformly on the nanoparticles.

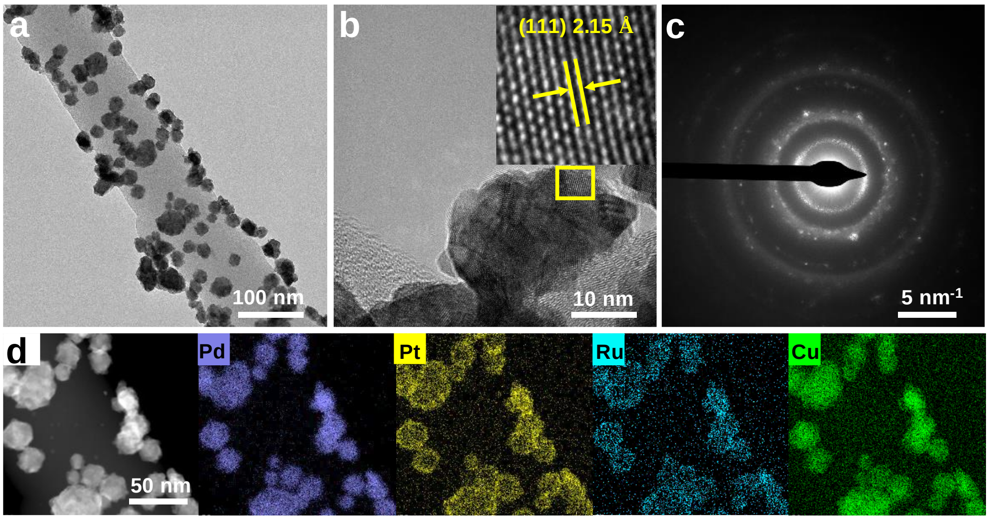

Supplementary Fig. 13 The TEM characterization of PdPtRuCu/CNFs. **a** TEM morphology. **b** HRTEM image. **c** Selective electron diffraction pattern. **d** EDS mapping. The EDS mapping indicates that the Pd, Pt, Ru, and Cu elements distribute uniformly on the nanoparticles.

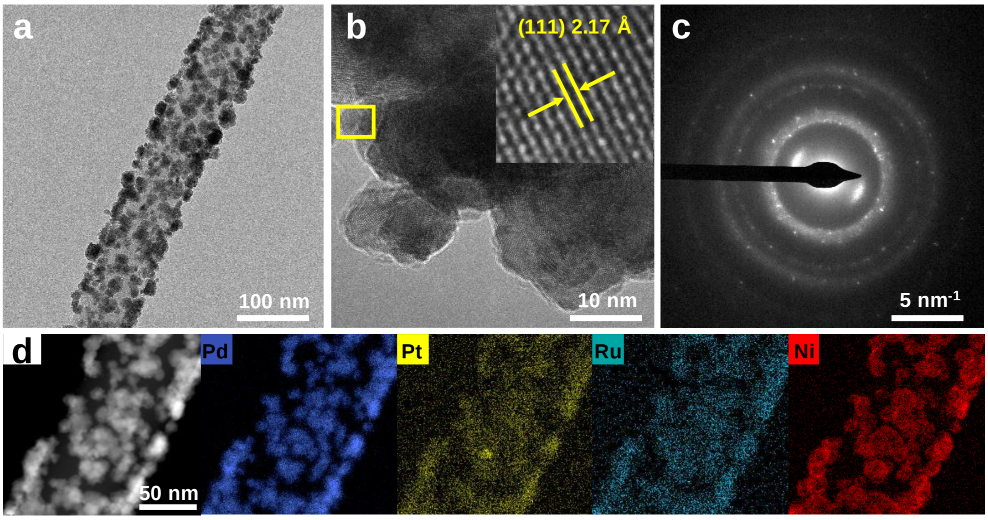

Supplementary Fig. 14 The TEM characterization of PdPtRuNi/CNFs. **a** TEM morphology. **b** HRTEM image. **c** Selective electron diffraction pattern. **d** EDS mapping. The EDS mapping indicates that the Pd, Pt, Ru, and Ni elements distribute uniformly on the nanoparticles.

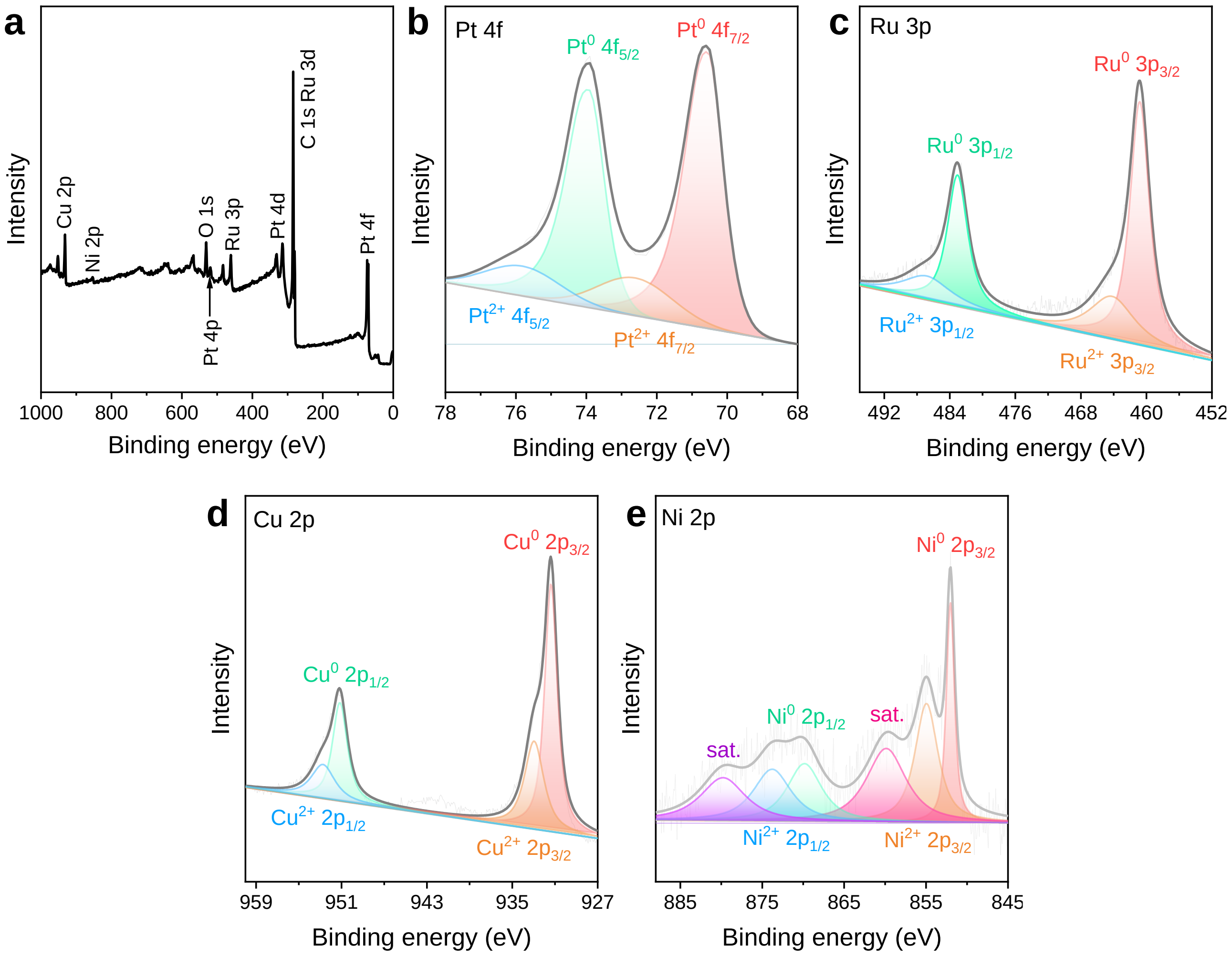

Supplementary Fig. 15 The XPS results of PtRuCuNi/CNFs. **a** Survey spectra. **b** Pt 4*f*, **c** Ru 3*p*, **d** Cu 2*p*, and **e** Ni 2*p* high-resolution spectra. The Pt, Ru, Cu exist mainly in their zero-valence states, the zero-valence state and oxidation state coexist for Ni.

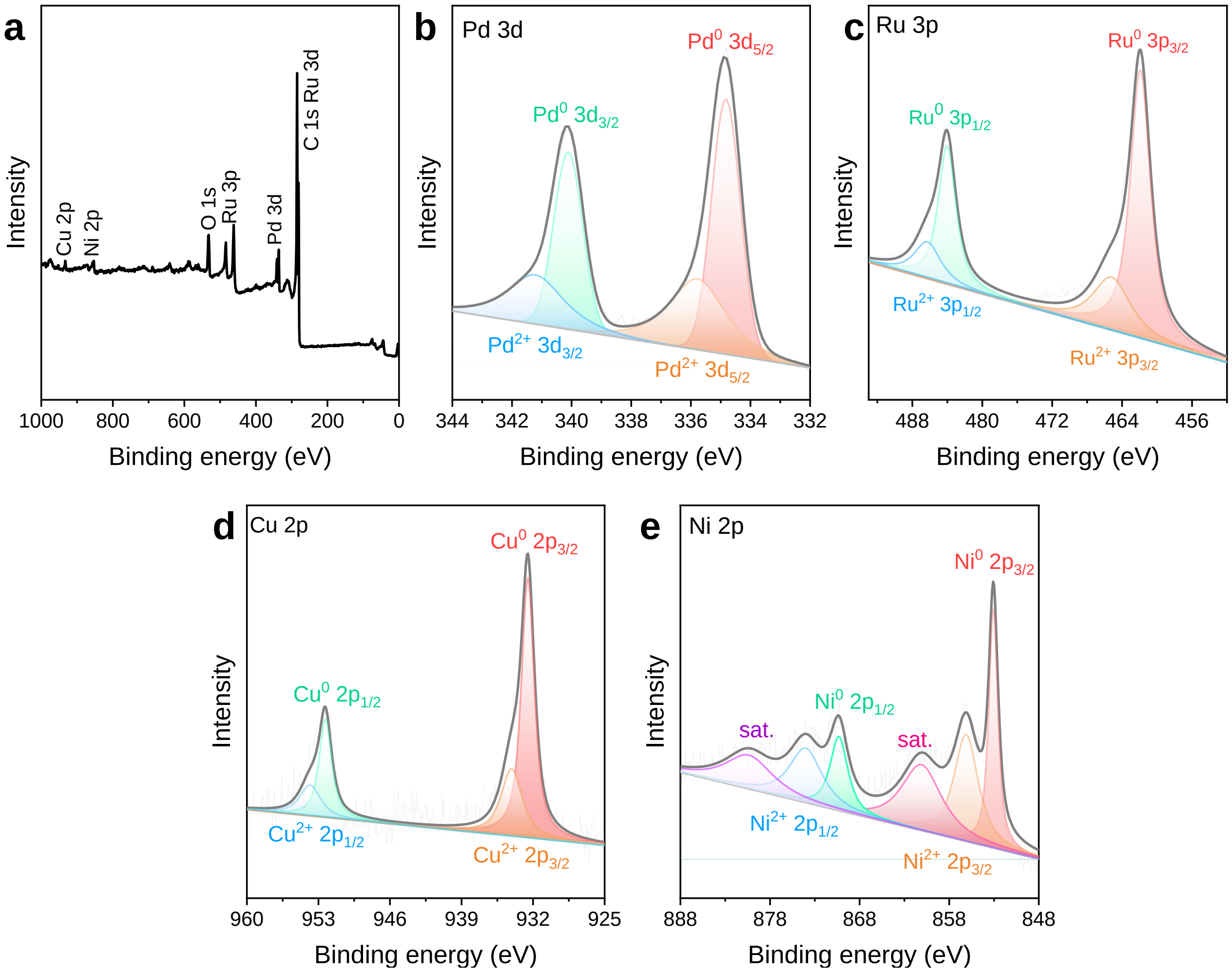

Supplementary Fig. 16 The XPS results of PdRuCuNi/CNFs. **a** Survey spectra. **b** Pd 3*d*, **c** Ru 3*p*, **d** Cu 2*p*, and **e** Ni 2*p* high-resolution spectra. The Pd, Ru, Cu exist mainly in their zero-valence states, the zero-valence state and oxidation state coexist for Ni.

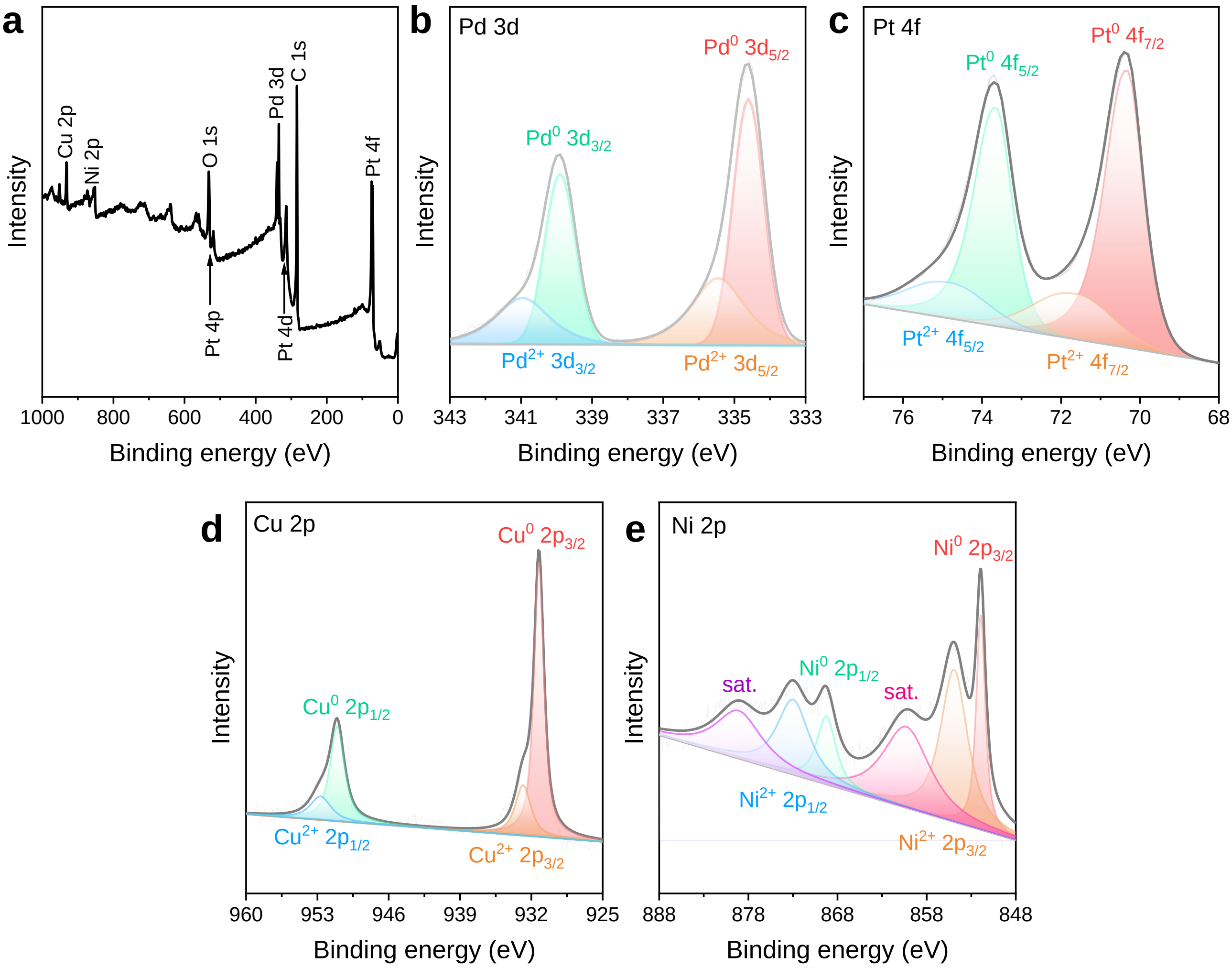

Supplementary Fig. 17 The XPS results of PdPtCuNi/CNFs. **a** Survey spectra. **b** Pd 3*d*, **c** Pt 4*f*, **d** Cu 2*p*, and **e** Ni 2*p* high-resolution spectra. The Pd, Pt, Cu exist mainly in their zero-valence states, the zero-valence state and oxidation state coexist for Ni.

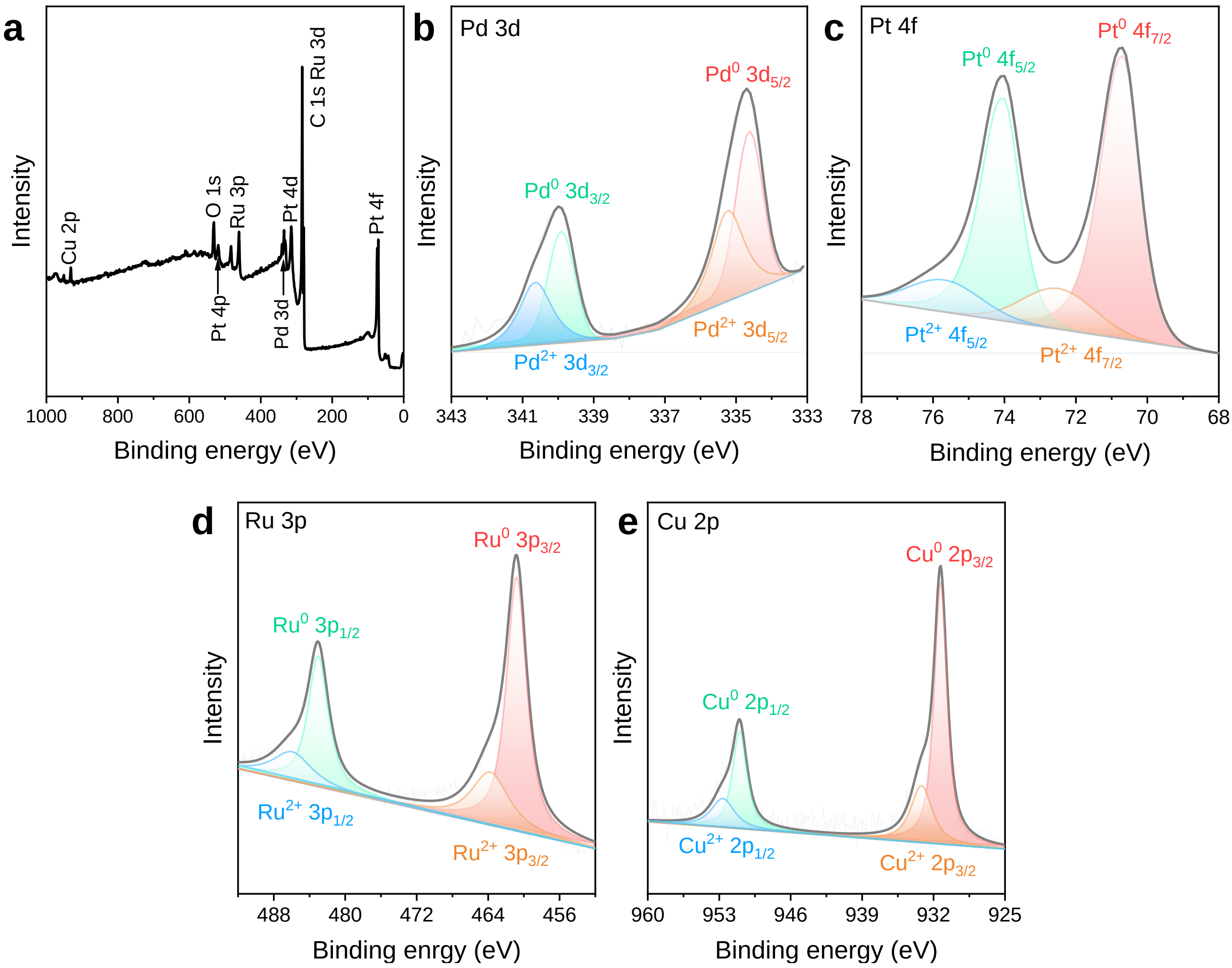

Supplementary Fig. 18 The XPS results of PdPtRuCu/CNFs. **a** Survey spectra. **b** Pd 3*d*, **c** Pt 4*f*, **d** Ru 3*p*, and **e** Cu 2*p* high-resolution spectra. The Pd, Pt, Ru and Cu exist mainly in their zero-valence states.

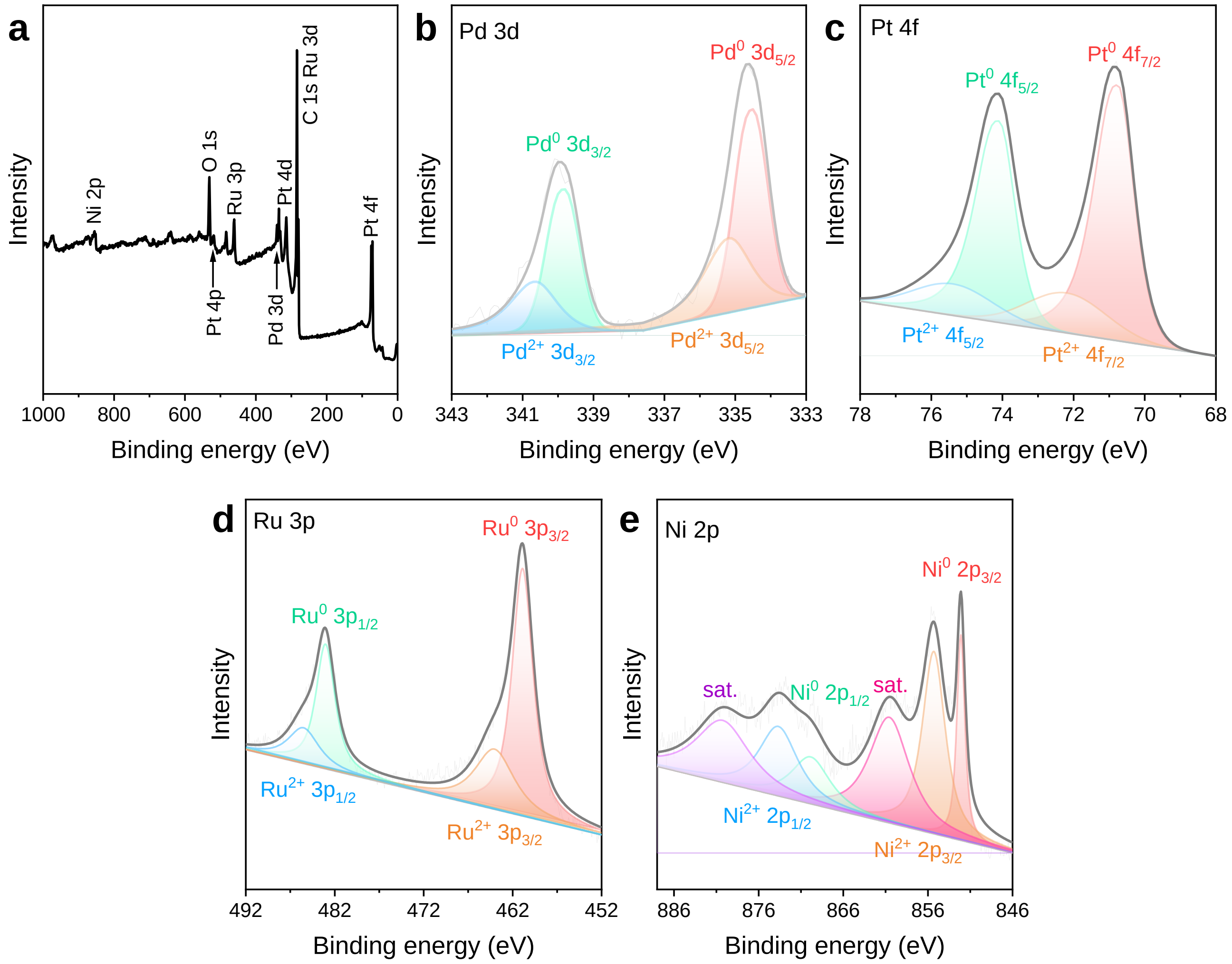

Supplementary Fig. 19 The XPS results of PdPtRuNi/CNFs. **a** Survey spectra. **b** Pd 3*d*, **c** Pt 4*f*, **d** Ru 3*p*, and **e** Ni 2*p* high-resolution spectra.The Pd, Pt, Ru exist mainly in their zero-valence states, the zero-valence state and oxidation state coexist for Ni.

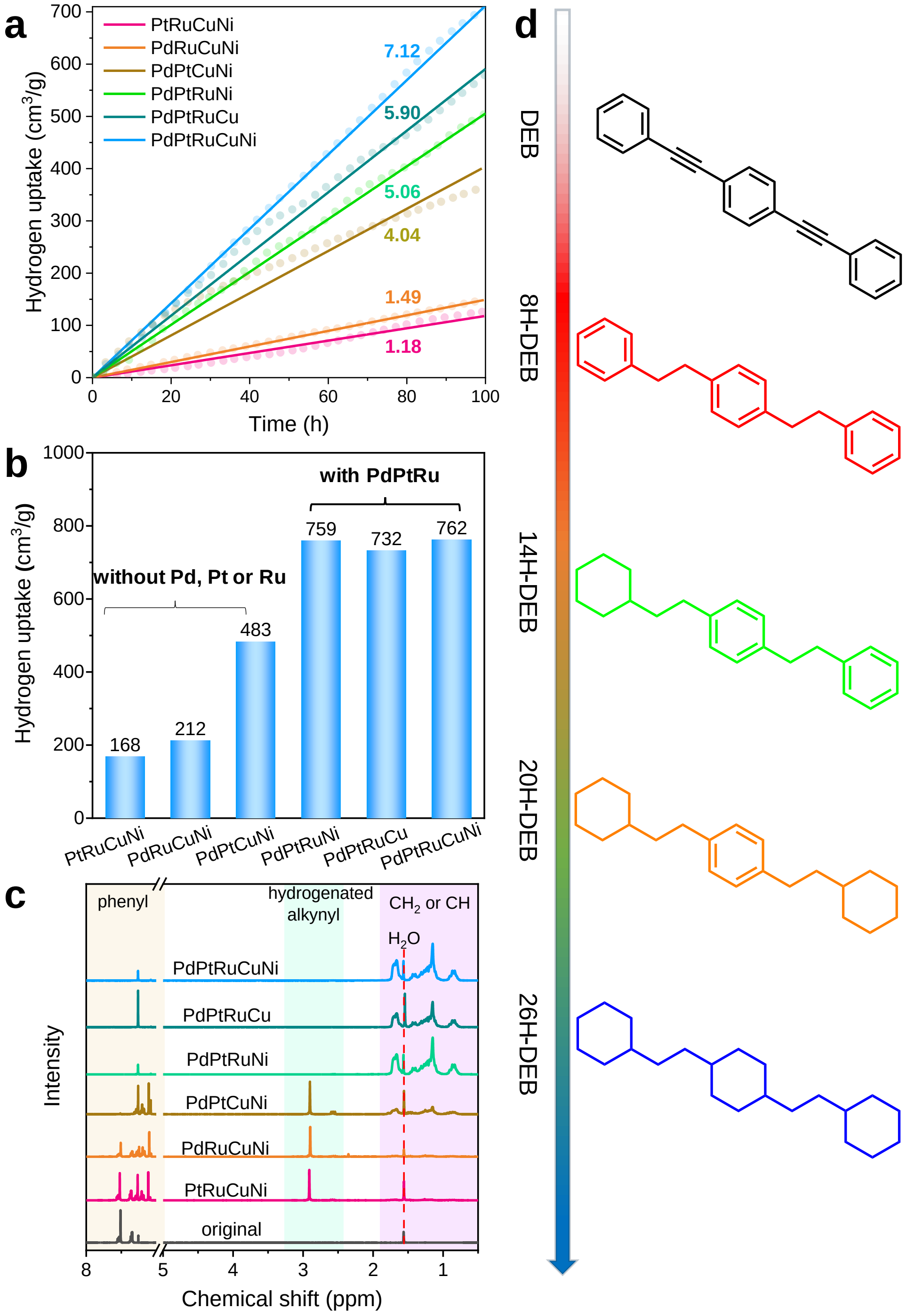

Supplementary Fig. 20 The hydrogenation of DEB catalyzed by quaternary and quinary nanocatalysts. a The hydrogen uptake curves (dot line) and reaction rates (slopes) of DEB with time. **b** The maximum hydrogen uptake capacities. **c** ^{1}H NMR spectra of DEB before and after hydrogenation. **d** The transformation of the DEB molecule from the original structure to the final hydrogenated products catalyzed by PdPtRu-contained catalysts.

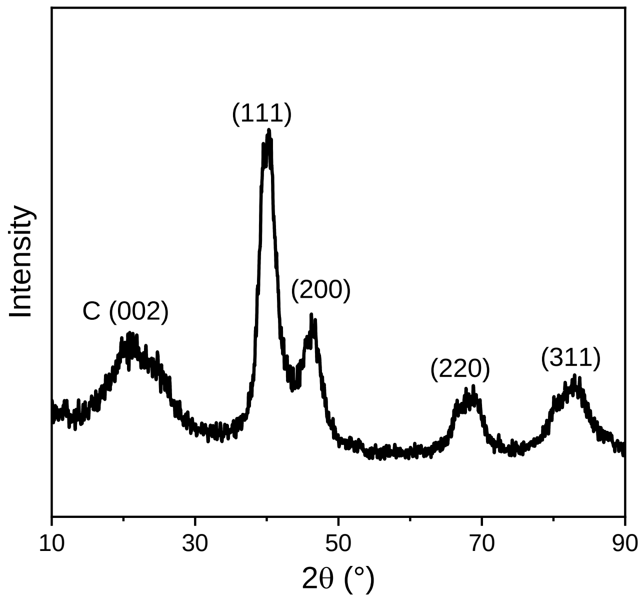

Supplementary Fig. 21 The XRD patterns of PdPtRu/CNFs catalyst. The PdPtRu catalyst is a single-phase alloy.

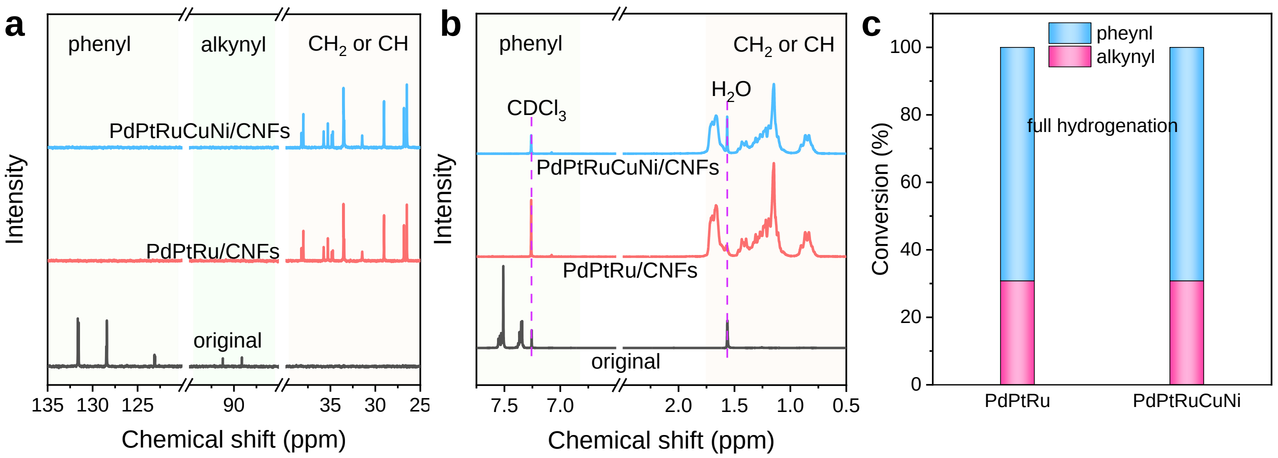

Supplementary Fig. 22 a H[1], and **b** ^{13}C NMR spectra of DEB before and after hydrogenation. **c** The conversion of C-C unsaturated bonds (alkynyl and phenyl groups) in the hydrogenated DEB molecules.

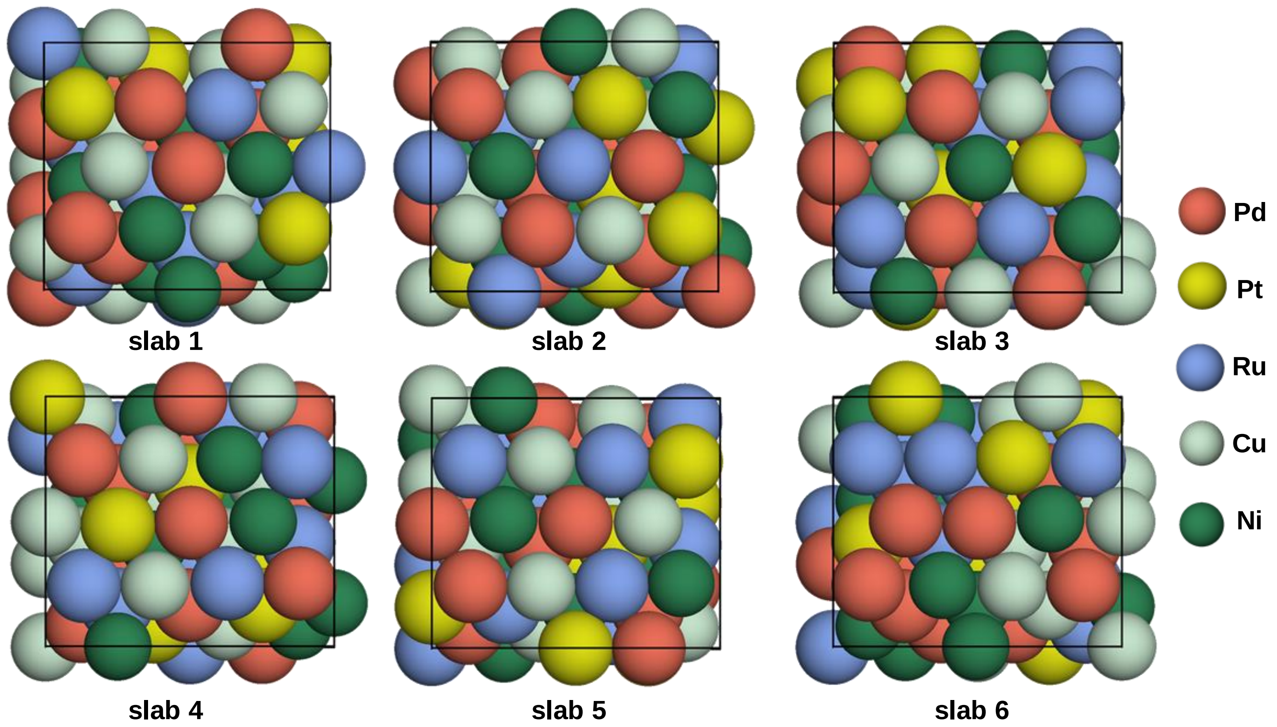

Supplementary Fig. 23 The six random slabs constructed for PdPtRuCuNi HEA (111). Slab 1-slab 6 with different atomic arrangement to investigate the effect of local atomic environment on hydrogenation.

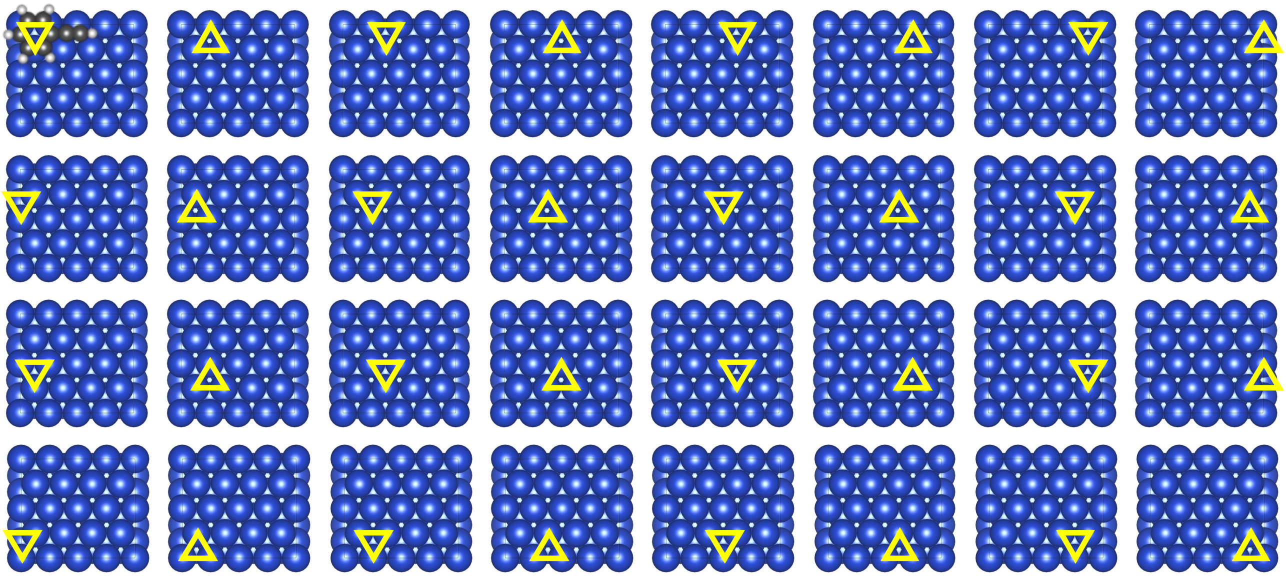

Supplementary Fig. 24 Illustration of the adsorption configurations of PhA. The top-left corner shows that the initial adsorption arrangement positions the phenyl group above a 3-fold hollow site, with the acetylenic group forming di-μ bonding. This setup aligns with the optimal adsorption configurations identified in prior studies and validated through our tests. The 32 adsorption configurations considered for each slab in our work are presented, with the hollow sites—where the phenyl group resides—highlighted by the yellow triangles.

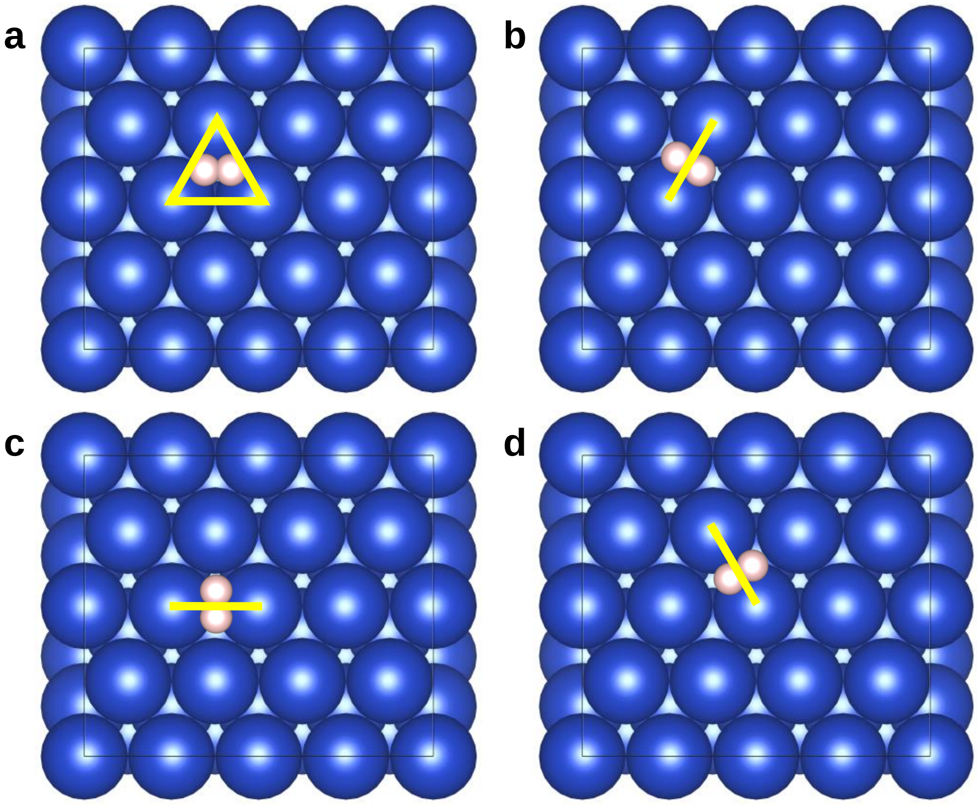

Supplementary Fig. 25 Illustration of the adsorption configurations of H_2. The adsorption configuration with one H_2 molecule positioned above the (**a**) hollow and (**b-d**) bridge sites is demonstrated. On our (111) surfaces, there are 32 hollow sites, consistent with those illustrated for PhA in Supplementary Figure 2, accompanied by 48 bridge sites.

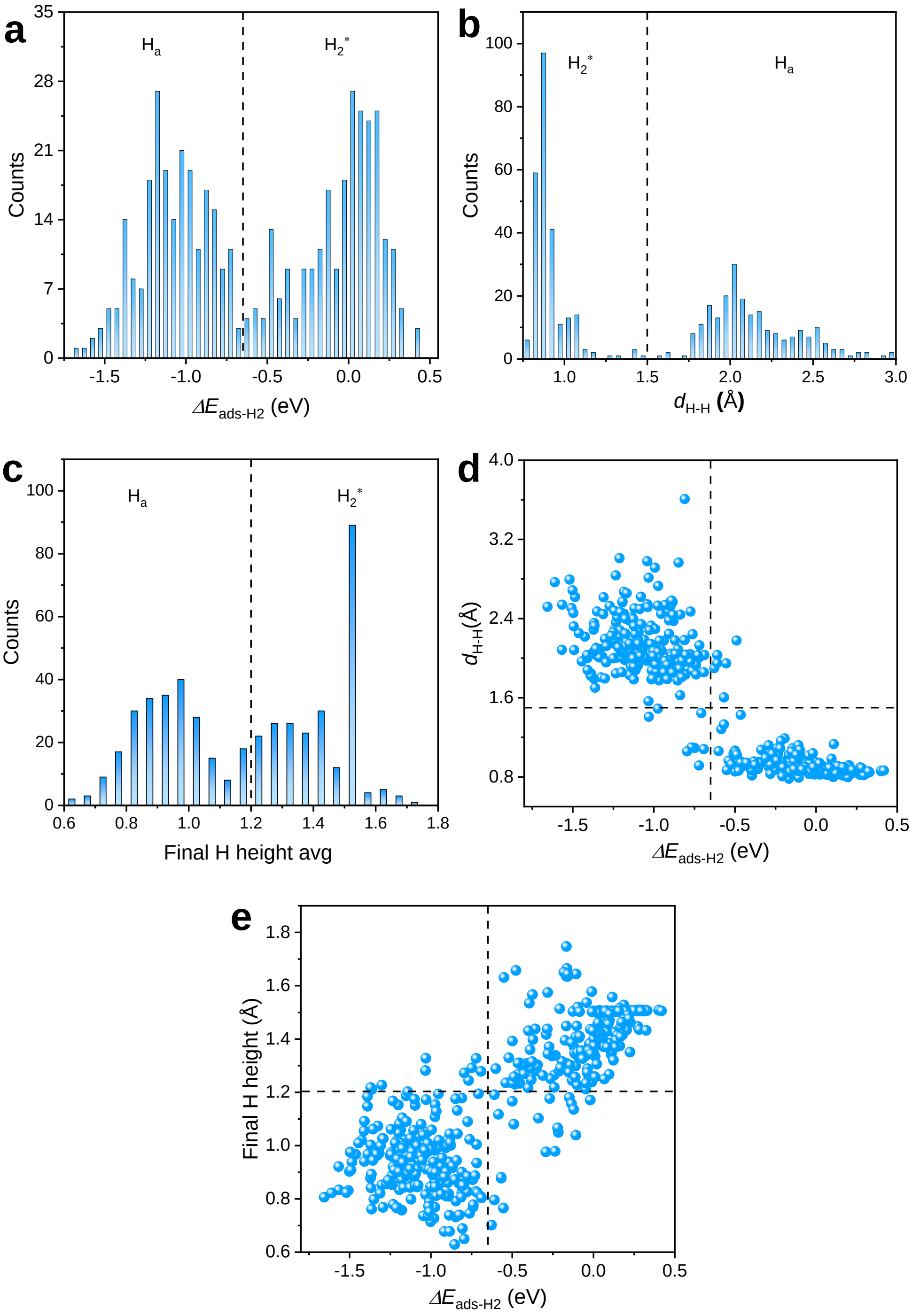

Supplementary Fig. 26 The absorption of H_2 molecules on the surface of PdPtRuCuNi HEA (111) calculated on 192 hollow- and 288 bridge-sites (480 sites in all). **a** The absorption energy. **b** The H-H bond distance activated by PdPtRuCuNi HEAs. **c** The distribution of final H heights to the surface of PdPtRuCuNi HEA(111). **d**,**e** The relationship of final H-H distance and H height with absorption energy.

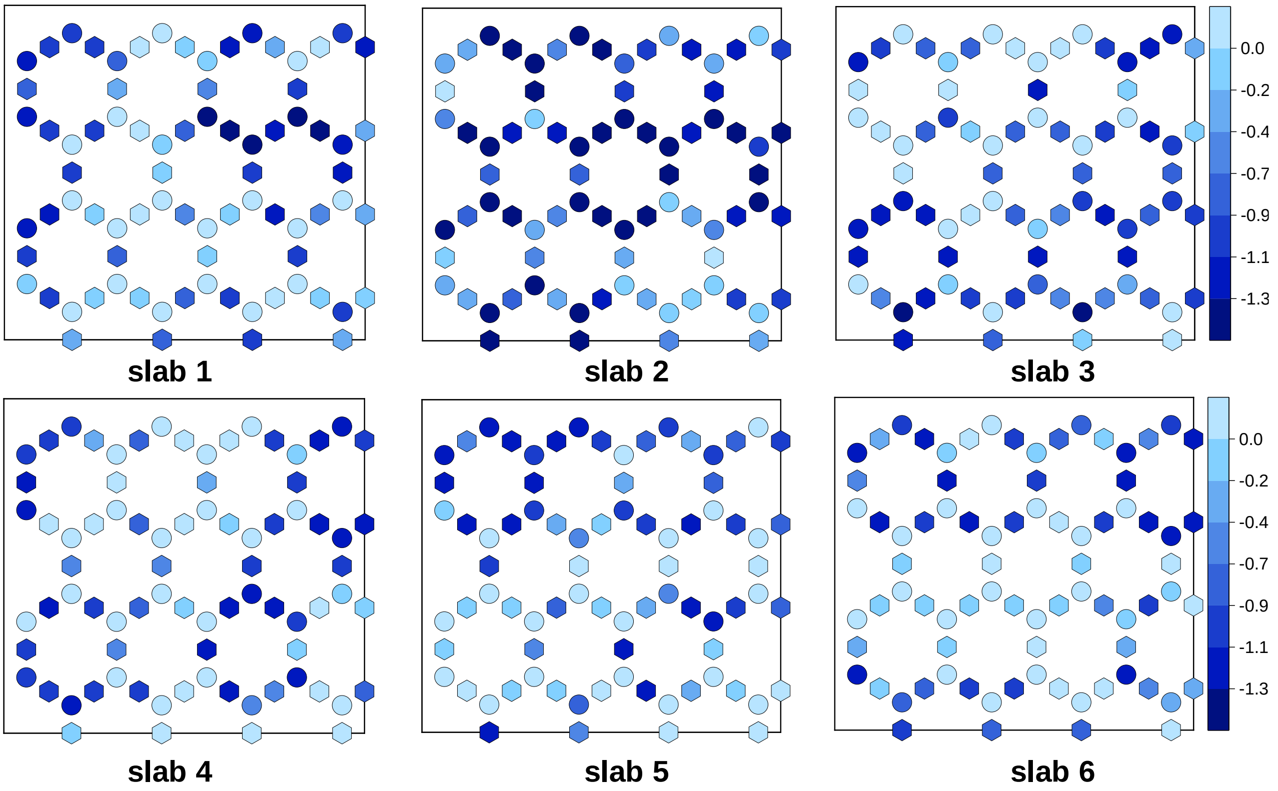

Supplementary Fig. 27 The spatial variation of ΔE_{ads-H_2} on the six different slabs of PdPtRuCuNi HEA (111). The circles represent the hollow sites, and hexagons represent the bridge sites. The surface atomic arrangements see **Supplementary Fig. 23**. Every slab has 32 hollow sites and 48 bridge sites for H_2, the color of sites is darker, indicating the stronger adsorption. Every slab has the sites favorable for hydrogen absorption.

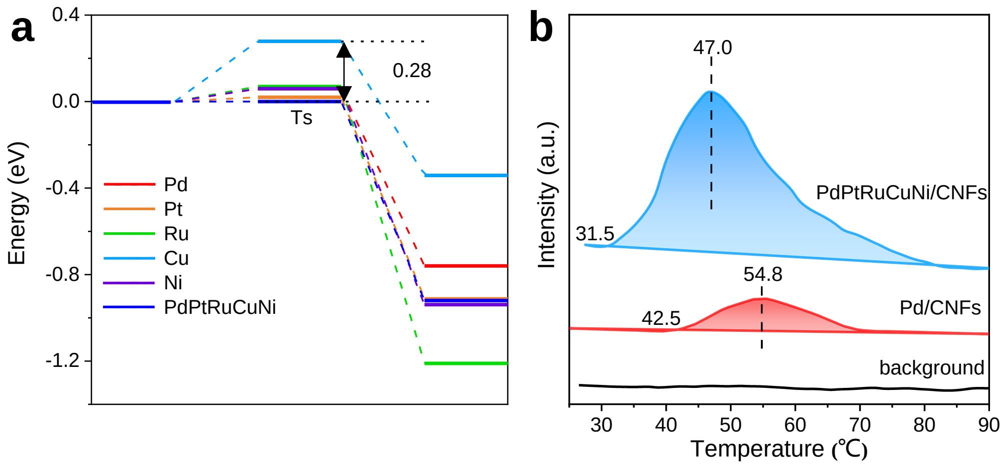

Supplementary Fig. 28 a The dissociated energies of H_2 on the (111) surface of PdPtRuCuNi HEA, Pd, Pt, Ru, Cu, and Ni catalysts. **b** TPD of H_2 from Pd/CNFs and PdPtRuCuNi/CNFs catalysts. The PdPtRuCuNi HEAs display the lowest activated energy for H_2 and an appropriate binding energy for H species. The desorption of H_2 from Pd/CNFs occurs in a single state with a maximum desorption peak located at 54.8 °C, while ~47 °C for PdPtRuCuNi HEAs, decreased about 7.8 °C, and a broader desorption peak of H_2 from HEA is appeared, demonstrating that alloying multiple metallic elements into individual nanoscale products can create the multi-site adsorption and the individual, isolated Pd atoms both assist adsorption and spillover of H species onto HEA by acting as the dissociation site for H_2 and, as would be expected from microscopic reversibility, serve as low-barrier exit routes for H_2 during the desorption process.

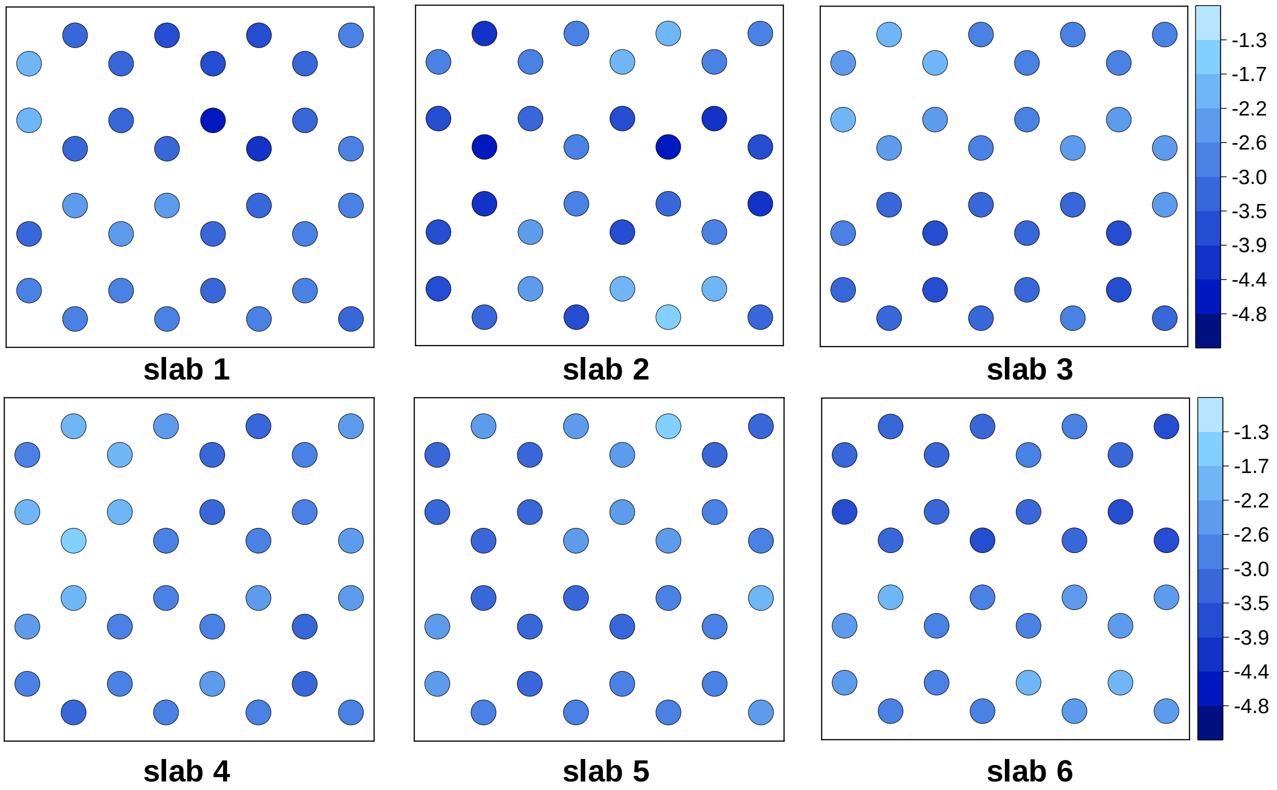

Supplementary Fig. 29 The spatial variation of $\Delta E_{\text{ads-PhA}}$ on the six different slabs of PdPtRuCuNi HEA (111). The surface atomic arrangements see **Supplementary Fig. 23**. Every slab has 32 hollow sites to absorb the PhA molecules, the color of sites became darker, indicating the adsorption of PhA more stonger. Every slab has the sites favorable for PhA absorption.

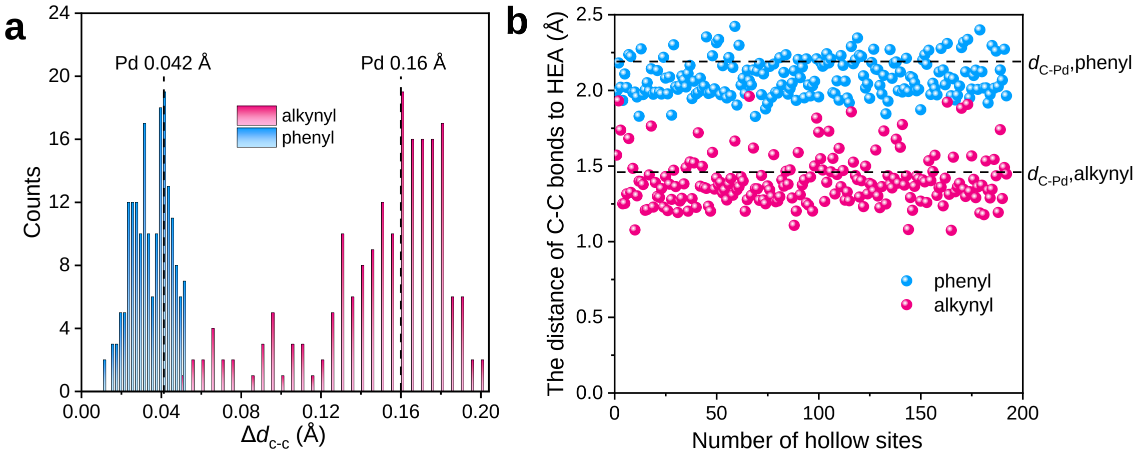

Supplementary Fig. 30 a The increased C-C bond distance of alkynyl and phenyl groups activated by Pd (line) and PdPtRuCuNi HEA (histogram) catalysts. **b** The height of alkynyl and phenyl groups to the surface of Pd (111) and PdPtRuCuNi HEA (111) after activated.

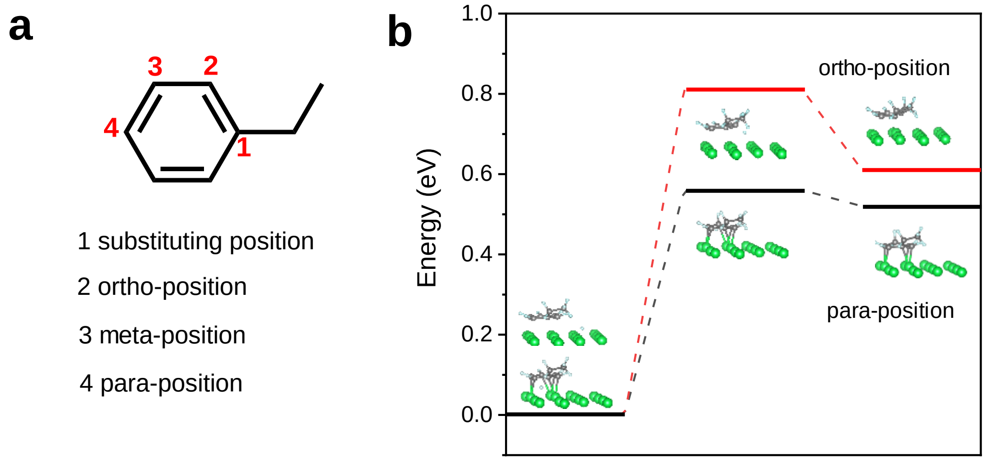

Supplementary Fig. 31 The hydrogenation reaction barrier of the first H_a added to different positions in the phenylethane on the Ru (111) surfaces. The substituting carbon (1) and the carbon in the meta-position (3) are without transition state in the hydrogenation process calculated by CI-NEB, while the carbon located at ortho- (2) and para-positions (4) have the possibility of preferential hydrogenation. Compared with the carbon in the ortho-position, the carbon in the para-position has the lowest hydrogenation reaction barrier. Therefore, we use para-C to calculate the first hydrogenation reaction barrier of phenylethane on the Pd (111), Ru (111), and PdPtRuCuNi HEA (111) surfaces.

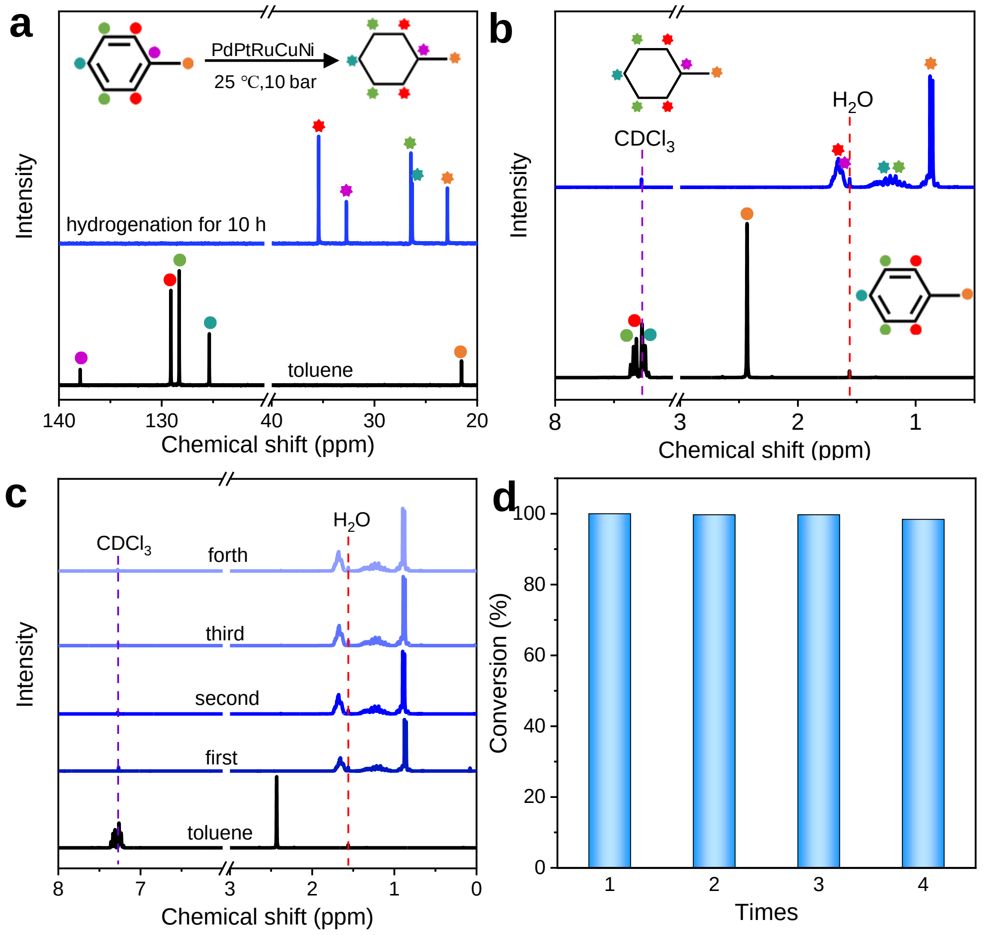

Supplementary Fig. 32 a ^{13}C and **b** ^{1}H NMR spectra of toluene before and after hydrogenation by PdPtRuCuNi HEA/CNFs, **c** ^{1}H NMR spectra of toluene catalyzed by the same PdPtRuCuNi HEAs with four times continuously, **d** The stable catalytic activity of PdPtRuCuNi HEAs for toluene. Reaction condition: 40 mg PdPtRuCuNi HEA/CNFs, 3 mL toluene, 25 °C, 10 bar H_2, 10 h.

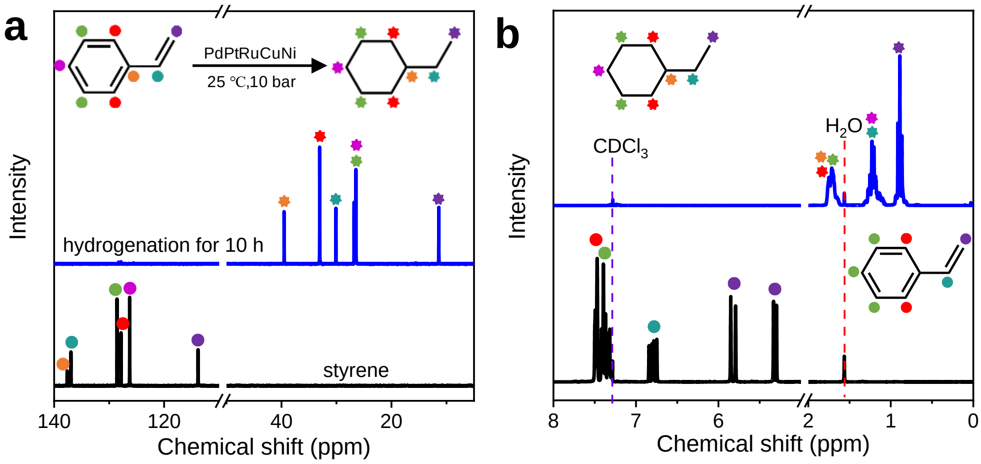

Supplementary Fig. 33 a ^{13}C and **b** ^{1}H NMR spectra of styrene before and after hydrogenation by PdPtRuCuNi HEA/CNFs. Reaction condition: 40 mg PdPtRuCuNi HEA/CNFs, 3 mL styrene, 25 °C, 10 bar H_2, 10 h.

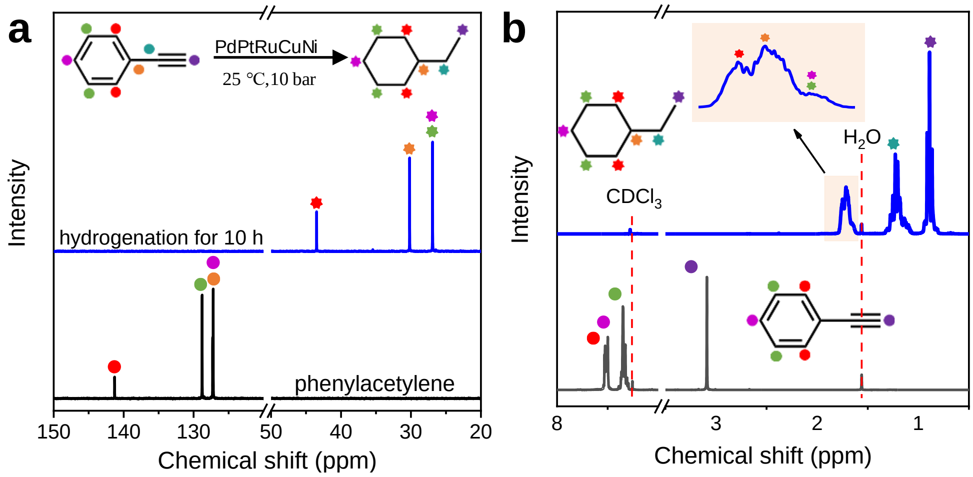

Supplementary Fig. 34 a ^{13}C and **b** ^{1}H NMR spectra of phenylacetylene before and after hydrogenation by PdPtRuCuNi HEA/CNFs. Reaction condition: 40 mg PdPtRuCuNi HEA/CNFs, 3 mL phenylacetylene, 25 °C, 10 bar H_2, 10 h.

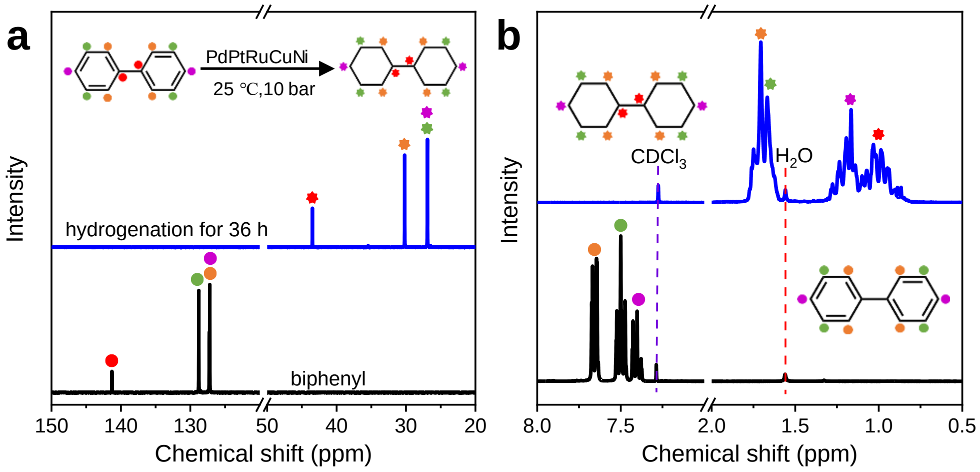

Supplementary Fig. 35 a ^{13}C and **b** ^{1}H NMR spectra of biphenyl before and after hydrogenation by PdPtRuCuNi HEA/CNFs. Reaction condition: 10 mg PdPtRuCuNi HEA/CNFs, 30 mg biphenyl, 25 °C, 10 bar H_2, 36 h.

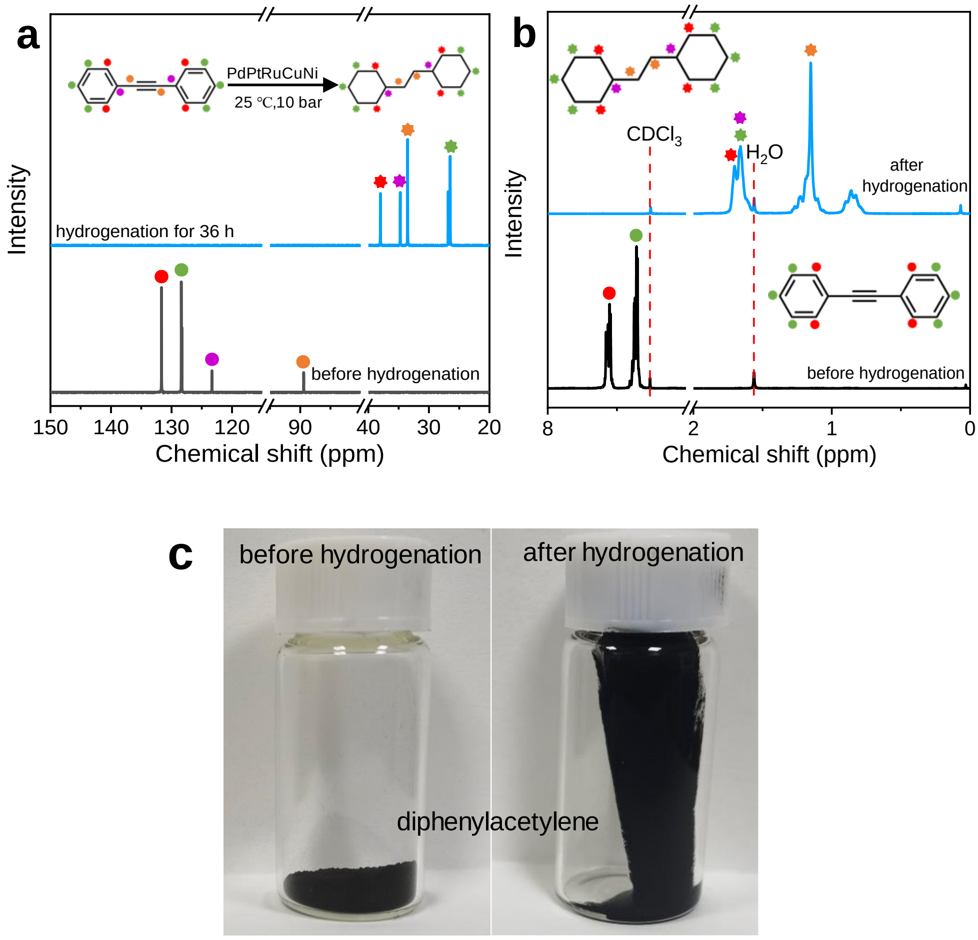

Supplementary Fig. 36 a ^{13}C and **b** ^{1}H NMR spectra of diphenylacetylene before and after hydrogenation by PdPtRuCuNi HEA/CNFs. **c** The diphenylacetylene/PdPtRuCuNi/CNFs composite changes from powder to melt after hydrogenation. Reaction condition: 10 mg PdPtRuCuNi HEA/CNFs, 30 mg diphenylacetylene, 25 °C, 10 bar H_2, 36 h.

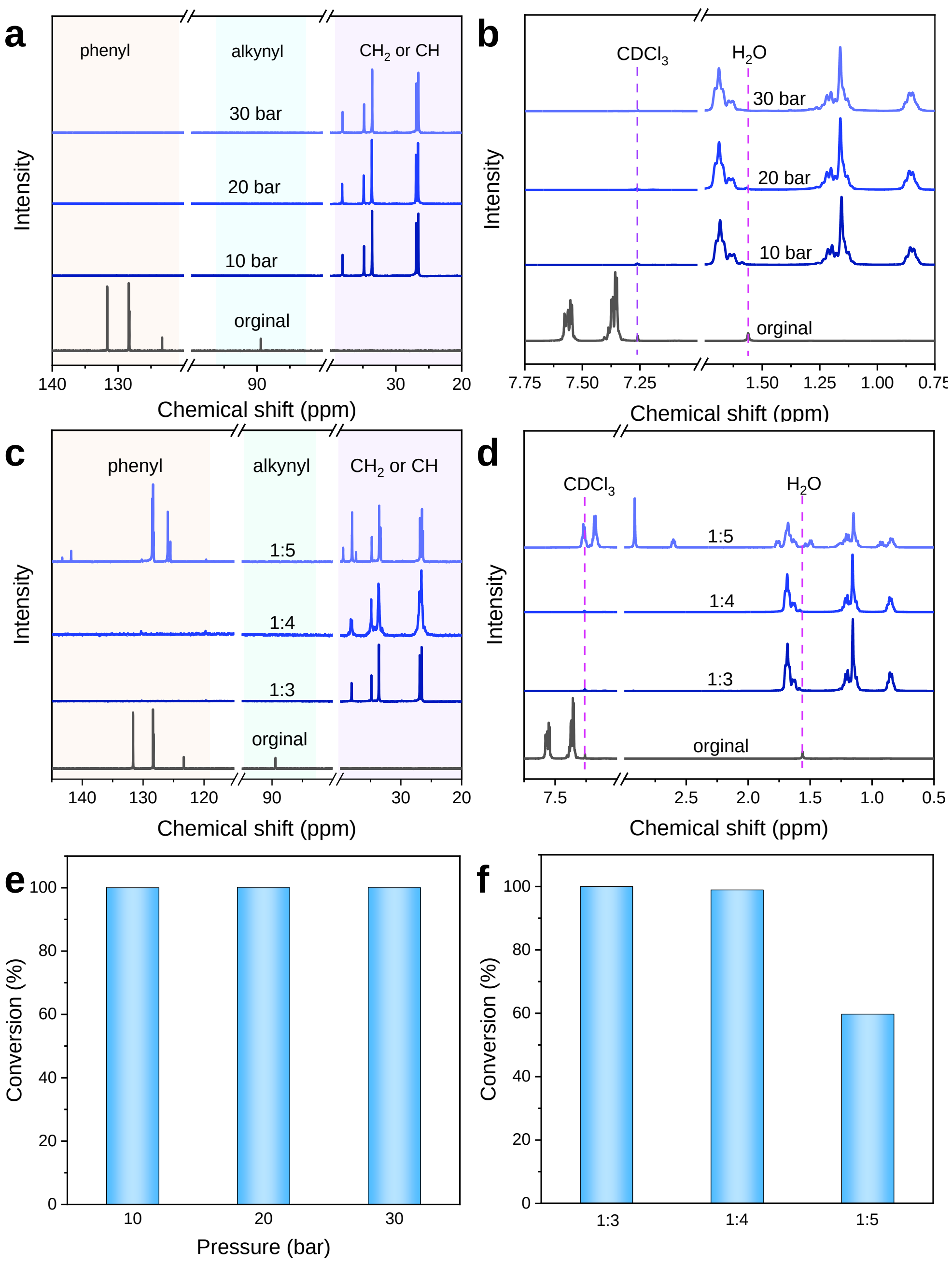

Supplementary Fig. 37 The diphenylacetylene were catalyzed by PdPtRuCuNi/CNFs at different hydrogen pressures (10 bar, 20 bar, and 30 bar) and amounts of catalyst (1:3,1:4, and 1:5). a ^{13}C and **b** ^{1}H NMR spectra of diphenylacetylene before and after hydrogenation. **e** The conversion at different hydrogen pressure. **c** ^{13}C and **d** ^{1}H NMR spectra of diphenylacetylene before and after hydrogenation. **f** The conversion at different amount of catalyst.

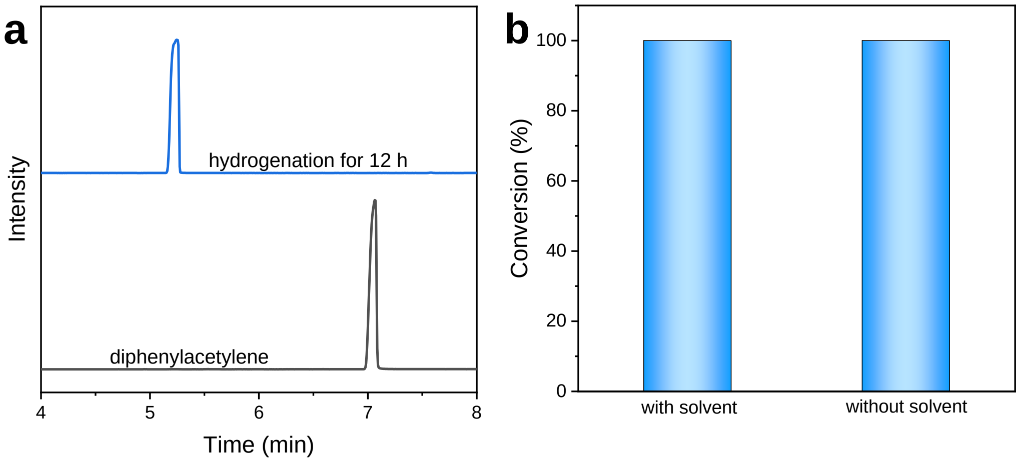

Supplementary Fig. 38 The conversion of diphenylacetylene catalyzed by PdPtRuCuNi/CNFs under liquid-phase compared with solid-phase. a The GC-MS results. **b** The conversion.

Tables:

Supplementary table 1. The atomic composition of PdPtRuCuNi/CNFs catalysts.

Sample	Pd	Pt	Ru	Cu	Ni	Load (wt%)	characterization
initial	21.3	12.4	20.4	23.9	22	55.2	ICP-OES
initial	20.4	13.3	20.8	24.0	21.5	---	SEM-mapping
after hydrogenation	21.6	13.9	20.7	22.5	21.3	---	SEM-mapping

Supplementary table 2. The (111) interplanar spacing of Pd, Pt, Ru, Cu, Ni single metal, and PdPtRuCuNi/CNFs catalysts.

Crystal facets	PdPtRuCuNi (d/Å)	Pd (d/Å)	Pt (d/Å)	Ru (d/Å)	Cu (d/Å)	Ni (d/Å)
(111)	2.20	2.25	2.29	2.21	2.09	1.99

Supplementary table 3. XRD data of Pd, Pt, Ru, Cu, Ni single metals and PdPtRuCuNi/CNFs catalysts.

Crystal facets	PdPtRuCuNi/CNFs (2 θ/°)	Pd (2 θ/°)	Pt (2 θ/°)	Ru (2 θ/°)	Cu (2 θ/°)	Ni (2 θ/°)
(111)	40.7	40.0	39.3	40.8	43.3	45.5
(200)	46.9	46.5	45.7	47.4	50.4	53.0
(220)	68.9	67.9	66.6	69.3	74.1	78.3
(311)	83.0	81.8	80.1	83.7	89.9	---

Supplementary table 4. EXAFS fitting parameters at the Cu and Ni K-edge, Pt L3-edge (S_0^2=0.90*)

Sample	Path	C.N.	R (Å)	$\sigma^2\times10^3$ (Å^2)	ΔE (eV)	R factor
Cu foil	Cu-Cu	12*	2.54±0.01	10.6±2.2	6.7±0.4	0.002
Cu	Cu-Cu	6.2±5.8	2.55±0.04	10.9±12.2	1.9±1.9	0.020
	Cu-Pd	2.0±0.7	2.56±0.06	5.0±5.9		
Ni foil	Ni-Ni	12*	2.48±0.01	6.2±0.2	7.4±0.3	0.001
Ni	Ni-Ni	3.3±0.7	2.45±0.01	6.8±1.7	-8.8±1.1	0.010
	Ni-Pd	1.7±1.0	2.64±0.05	17.7±9.5	11.8±1.5	
Pt foil	Pt-Pt	12*	2.77±0.01	4.8±0.2	3.6±0.5	0.001
Pt	Pt-Cu	6.4±2.7	2.60±0.02	16.1±5.5	4.0±1.7	0.004
	Pt-Pd	4.8±1.1	2.71±0.03	14.0±4.2		

C.N.: coordination numbers; *R*: bond distance; σ^2: Debye-Waller factors; Δ*E*: the inner potential correction. *R* factor: goodness of fit. * fitting with fixed parameter.

Supplementary table 5. The total load and mono-metal load of catalysts.

samples	Pd (wt%)	Pt (wt%)	Ru (wt%)	Cu (wt%)	Ni (wt%)	Toal (wt%)
PdPtRuCuNi/CNFs	12.5	15.0	12.1	8.3	7.3	55.2
PtRuCuNi/CNFs		17.8	11.3	9.3	6.9	45.3
PdRuCuNi/CNFs	16.3		12.0	10.2	9.0	47.5
PdPtCuNi/CNFs	14.9	16.2		10.9	7.4	49.4
PdPtRuNi/CNFs	16.2	13.5	12.9		8.2	50.8
PdPtRuCu/CNFs	9.8	19.2	7.2	10.4		46.6
PdPtRu/CNFs	19.5	20.2	13.9			53.6
Ru/C			4.6			4.6
Pd/C	4.9					4.9
Pd/CNFs	60.3					60.3

Supplementary table 6. The absorption properties of H_2 on Pd (111) surface.

site	hollow	bridge1	bridge2	bridge3
optimized structures	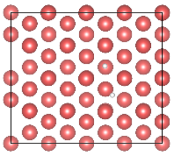	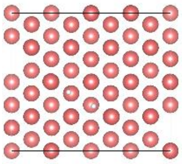	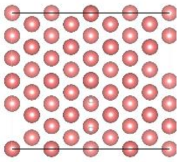	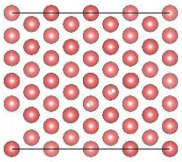
Absorption energy (eV)	0.97	0.95	0.96	0.95
$d_{H\text{-}H}$ (Å)	2.13	1.92	1.93	1.93
Final H height (avg, Å)	1.06	0.99	0.93	0.97

Supplementary table 7. Catalytic hydrogenation of aromatic substrates over PdPtRuCuNi /CNFs catalyst at 25 °C and 10 bar H_2.

Entry	Catalyst	Solvent	Reactive condition	Ratio (catalyst/substrate)	Yield/Conversion (%)	Ref.
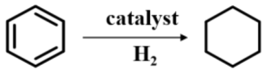						
1	Ru/CUA-N	n-hexane	80 °C, 30 bar	1:100 (wt)	---	1
2	Ru/Nb_2O_5-nC18PA	decalin and water	80 °C, 12 bar	1:1(wt)	91.5%	2
3	$Cu_1Co_5Ce_5O_x$	hexane	100 °C, 5 bar	100 mg:1.25 mmol	99%	3
4	Rh(0)@HAp(0.5wt%)	without	25 °C, 3 bar	100 mg:1 mL	99%	4
5	$LiAlH_4/Fe^0$ (2.5 mol%)	without	120 °C, 50 bar	---	98%	5
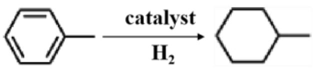						
6	10%Ru/Al_2O_3	isopropanol	30 °C, 10 bar	3:10 (wt)	100%	6
7	3.3%Ru/C-silica	without	110 °C, 40 bar	1:10000 (mol, Ru:toluene)	99.8%	7
8	Ru−B/MIL-53(AlCr)	ethanol	30 °C, 10 bar	1:595 (mol, Ru:toluene)	53%	8
9	$Ti^{III}{}_2Ti^{IV}{}_6$-BDC-CoH (0.05%)	---	120 °C, 50 bar	---	76%	9
10	$Cu_1Co_5Ce_5O_x$	hexane	100 °C, 5 bar	100 mg:1.25 mmol	99%	3
11	Ru/CNTs	without	80 °C, 40 bar	50 mg:3 mL	99.97%	10
12	Ni/Al_2O_3	n-hexane	150 °C, 40 bar	---	98%	11
13	$Pt_{0.051}Cu_{5.7}$	without	120 °C, 1bar	---	---	12
14	Rh	without	200 °C, 1bar	---	100%	13

15	Pd/SiO_2	n-decane	120 °C, 20 bar	1:2 (wt)	---	14
16	$Ir\text{-}HCP\text{-}CH_3$	hexane	120 °C, 20.7 bar	40 mg:1 mmol	---	15
17	Ni	n-hexane	140 °C, 50 bar	25 mg:2 mL	100%	16
18	Pt/S-1	n-hexane	150 °C, 15 bar	1 g:0.011 mol	99.9%	17
19	$AliBu_3/Fe^0$(2.5mol%)	without	150 °C, 50 bar	---	99%	5
20	$Ru/NiCo/Ni(OH)_2\text{-}Co(OH)_2/C$	without	60 °C, 45.5 bar	0.1 g:20 mL	100%	18
21	PdPtRuCuNi/CNFs	without	25 °C, 10 bar	40 mg:3 mL	100%	**this work**

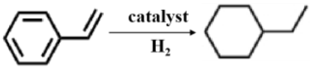

22	Ru@ saddle-shaped nanographenes	tetrahydrofuran	50 °C, 10 bar	1:20.8 (mol, Ru:styrene)	94%	19
23	$Cu_1Co_5Ce_5O_x$	hexane	100 °C, 5 bar	100 mg:1.25 mmol	99%	3
24	PdPtRuCuNi/CNFs	without	25 °C, 10 bar	40 mg:3 mL	98.6%	**this work**

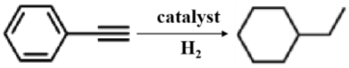

25	$Cu_1Co_5Ce_5O_x$	hexane	100 °C, 5 bar	100 mg:1.25 mmol	99%	3
26	PdPtRuCuNi/CNFs	without	25 °C, 10 bar	40 mg:3 mL	98.5%	**this work**

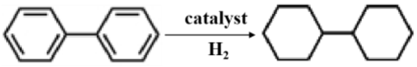

27	$10\%Ru/Al_2O_3$	isopropanol	30 °C, 10 bar	3:10 (wt)	100%	6
28	NiMoWS-0.70	hexadecane	300 °C, 55 bar	0.3:0.24 (wt)	47%	20
29	Rh/C (10wt%)	isopropanol	60 °C, 1 bar	1:10 (wt)	97%	21

30	Ru/SiO_2(1.5 wt%)	isopropanol	90 °C, 20 bar	1:11.8 (wt)	99.9%	22
31	PdPtRuCuNi/CNFs	without	25 °C, 10 bar	1:3 (wt)	100%	**this work**

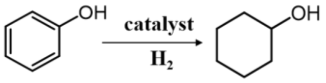

31	Ni/AC (10wt.%)	water	150 °C, 15 bar	0.3:1 (wt)	94.3%	23
32	Pd/NaY	ethanol	235 °C, 50 bar	0.1:1 (wt)	92.3%	24
33	Ru/Nb_2O_5-nC18PA	decalin and water	80°C, 12 bar	0.05:0.05 (wt)	93%	2
34	Ru/C (10wt.%)	isopropanol	60 °C, 5 bar	1:10 (wt)	92%	21
35	Ru@NDCs-800	water	25°C, 5 bar	20 mg:0.5 mmol	91%	25
36	Ru/MIL-101	ethanol	50°C, 5 bar	100 mg:2.5 mmol	40%	26

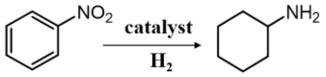

37	Ru/DMSN-Ph	IPA/water (95:5 v/v%)	100 °C, 30 bar	1:100 (mol)	92.4%	27
38	Ru-HDA	ethanol	80 °C, 30 bar	1:160 (mol, Ru:nitrobenzene)	83%	28
39	Ru/g–C_3N_4–H_2	tetrahydrofuran	130 °C, 50 bar	1:4 (wt)	97%	29
40	Ru/AC	isopropanol	90 °C, 60 bar	20 mg:1 mmol	85.4%	30

CUA-N: Al_2O_3 rich in pentacoordinate Al^{3+} centers with N_2 treatment

nC18PA: octadecyl phosphonic acid

HAp: hydroxyapatite

MIL-53 and MIL-101: metal-organic frameworks

HCP-CH_3: hyper-crosslinked porous polymer with CH_3 groups

NDCs-800: vulcan powder with heat treatment at 800 °C

DMSN: dendritic mesoporous silica nanospheres

HAD: hexadecylamine

Supplementary table 8. The hydrogen uptake properties of different organic hydrogen getters catalyzed by Pd-based catalysts at 25 °C.

Hydrogen getters	Pressure (bar)	Reaction time	Hydrogen uptake (cm^3/g)	Reactive groups	Ref.
Pd/C-DEB	0.013	20 h	216.0 [a]	alkynyl	31
	0-1.0 [c]	120 h	186 [a]	alkynyl	**this work**
Pd/rGO-DEB	0-1.0 [c]	20 h	178.0 [a]	alkynyl	32
Pd/GA-DEB	0-1.0 [c]	48 h	215.5 [a]	alkynyl	33
PdPtRuCuNi/CNFs-DEB	0-1.0 [c]	120 h	761 [a]	alkynyl and benzene ring	**this work**
PdPtRuCu/CNFs-DEB	0-1.0 [c]	120 h	759 [a]	alkynyl and benzene ring	**this work**
PdPtRuNi/CNFs-DEB	0-1.0 [c]	120 h	732 [a]	alkynyl and benzene ring	**this work**
DEB-Pd/C@silicone cast (4.3 wt% Pd-C/DEB)	0.001	600 h	3.0-6.0 [b]	alkynyl	34
DEB-Pd/C@silicone (3D) (4.3 wt% Pd-C/DEB)	0.001	600 h	6.0-12.0 [b]	alkynyl	34
DEB-Pd/C@silicone foam (40.0 wt% Pd-C/DEB)	0.013-0.067	1-5 h	72.0-81.9 [b]	alkynyl	35
DEB-Pd/C@PEO (30.0 wt% Pd-C/DEB)	2.0	10 h	14.0-102.1 [b]	alkynyl	36
DEB-Pd/C@SEBS(50.0 wt% Pd-C/DEB)	0-1.0 [c]	48 h	109.6 [a]	alkynyl	37

a—the hydrogen uptake capacity is measured directly.

b—estimated by the hydrogenation reaction conversion of DEB filled in the sample reported in the literature.

c—the hydrogen uptake experiments were performed using an automatic hydrogen uptake tester at 25 °C under 0-1 bar H_2. Twenty different equilibrium pressures at 0-1 bar (0.05 bar, 0.1 bar, 0.15 bar, 0.2 bar, up to 1 bar) were set in the hydrogen uptake measurement, and the sample was allowed to uptake hydrogen gas until the hydrogen pressure dropped to the pre-set value.

Supplementary References

1. Han, W. et al. Coordinatively unsaturated aluminum anchored Ru cluster for catalytic hydrogenation of benzene. J. Catal. **400**, 255-264 (2021).
2. Zhan, J. et al. Highly selective conversion of phenol to cyclohexanol over Ru/Nb_2O_5-nC18PA catalysts with increased acidity in a biphasic system under mild conditions. Green Chem. **24**, 1152-1164 (2022).
3. Chen, H. et al. Facile benzene reduction promoted by a synergistically coupled Cu-Co-Ce ternary mixed oxide. Chem. Sci. **11**, 5766-5771 (2020).
4. Zahmakıran, M., Román-Leshkov, Y. & Zhang, Y. Rhodium(0) nanoparticles supported on nanocrystalline hydroxyapatite: highly effective catalytic system for the solvent-free hydrogenation of aromatics at room temperature. Langmuir **28**, 60-64 (2011).
5. Knüpfer, C. Färber, C. Langer, J. & Harder, S. Lithium aluminium hydride and metallic iron: A powerful team in alkene and arene hydrogenation catalysis. Angew. Chem. Int. Edit. **62**, e202219016 (2023).
6. Jiang, W. et al. Catalytic hydrogenation of aromatic ring over ruthenium nanoparticles supported on α-Al_2O_3 at room temperature. Appl. Catal. B: Environ. **307**, 121137 (2022).
7. Su, F. et al. Thermally reduced ruthenium nanoparticles as a highly active heterogeneous catalyst for hydrogenation of monoaromatics. J. Am. Chem. Soc. **129**, 14213-14223 (2007).
8. Bi, H. et al. Ru-B nanoparticles on metal-organic frameworks as excellent catalysts for hydrogenation of benzene to cyclohexane under mild reaction conditions. Green Chem. **18**, 2216-2221 (2016).
9. Ji, P. et al. Titanium(III)-oxo clusters in a metal-organic framework support single-site Co(II)-hydride catalysts for arene hydrogenation. J. Am. Chem. Soc. **140**, 433-440 (2018).
10. Ma, Y. Huang, Y. Cheng, Y. Wang, L. & Li, X. Biosynthesized ruthenium nanoparticles supported on carbon nanotubes as efficient catalysts for hydrogenation of benzene to cyclohexane: An eco-friendly and economical bioreduction method. Appl. Catal. A: Gen. **484**, 154-160 (2014).
11. Cai, J. Zuo, L. Hao, C. Fu, Y. & Shen, J. Effect of ethylamines on the hydrogenation of toluene over supported nickel catalysts. Ind. Eng. Chem. Res. **57**(45): 15262-15267 (2018).
12. Oda, A. Fujita, T. Yamamoto, Y. Sawabe, K. & Satsuma, A. Breaking the structure-activity relationship in toluene hydrogenation catalysis by designing heteroatom ensembles based on a single-atom alloying approach. ACS Catal. **13**, 10026-10040 (2023).
13. Furukawa, S. et al. Remarkable enhancement in hydrogenation ability by phosphidation of ruthenium: specific surface structure having unique Ru ensembles. ACS Catal. **8**, 8177-8181 (2018).
14. Wu, D. et al. Surface molecular imprinting over supported metal catalysts for size-dependent selective hydrogenation reactions. Nat. Catal. **4**, 595-606 (2021).
15. Shi, S. et al. Selective hydrogenation via precise hydrogen bond interactions on catalytic scaffolds. Nat. Commun. **14**, 429 (2023).
16. Song, H. Wang, T. Jia, X. & Zhu, Y. Nickel hierarchical flower-like nanostructures: Controlled synthesis and catalytic hydrogenation of toluene. J. Alloy Compd. **747**, 523-529 (2018).
17. Zhang, X. et al. Study of the carbon cycle of a hydrogen supply system over a supported Pt catalyst: methylcyclohexane-toluene-hydrogen cycle. Catal. Sci. Technol. **10**, 1171-1181 (2020).
18. Zhu, L. et al. Nickel hydroxide-cobalt hydroxide nanoparticle supported ruthenium-nickel-cobalt

islands as an efficient nanocatalyst for the hydrogenation reaction. Chem. Cat. Chem. **10**, 1998-2002 (2018).
19. Cerezo-Navarrete, C. et al. Ruthenium nanoparticles canopied by heptagon-containing saddle-shaped nanographenes as efficient aromatic hydrogenation catalysts. Chem. Sci. **13**, 13046-13059 (2022).
20. Olivas, A. Gaxiola, E. Cruz-Reyes, J. Alvarez-Amparán, M. A. & Valdez, R. Transition-metal influence (Fe, Co, Ni, Cu) on the MoWS catalyst for biphenyl hydrogenation. Fuel Process. Technol. **204**, 106410 (2020).
21. Maegawa, T. et al. Efficient and practical arene hydrogenation by heterogeneous catalysts under mild conditions. Chem-Eur. J. **15**, 6953-6963 (2009).
22. Su, W. et al. Highly dispersed and ultra-small Ru nanoparticles deposited on silica support as highly active and stable catalyst for biphenyl hydrogenation. Mol.Catal. **508**, 111577 (2021).
23. Zhao, Z. et al. The influence of carbon supports and their surface modification on aqueous phase highly selective hydrogenation of phenol to cyclohexanol over different Ni/carbon catalysts. Carbon **213**, 118227 (2023).
24. Xia, H. et al. Tunable selectivity of phenol hydrogenation to cyclohexane or cyclohexanol by a solvent-driven effect over a bifunctional Pd/NaY catalyst. Catal. Sci. Technol. **11**, 1881-1887 (2021).
25. Cui, X. et al. Highly selective hydrogenation of arenes using nanostructured ruthenium catalysts modified with a carbon-nitrogen matrix. Nat. Commun. **7**, 11326 (2016).
26. Ertas, I. E. Gulcan, M. Bulut, A. Yurderi, M. & Zahmakiran, M. Metal-organic framework (MIL-101) stabilized ruthenium nanoparticles: Highly efficient catalytic material in the phenol hydrogenation. Micropor. Mesopor. Mat. **226**, 94-103 (2016).
27. Li, X. Ren, X. Guo, M. Li, W. & Yang, Q. Water accelerated activity of Ru NPs in sequential hydrogenation of nitrobenzene to cyclohexylamine. J. Catal. **413**, 546-553 (2022).
28. Axet, M. R. Conejero, S. & Gerber, I. C. Ligand effects on the selective hydrogenation of nitrobenzene to cyclohexylamine using ruthenium nanoparticles as catalysts. ACS Appl. Nano Mater. **1**, 5885-5894 (2018).
29. Yang, H. et al. Green and selective hydrogenation of aromatic diamines over the nanosheet Ru/g-C_3N_4-H_2 catalyst prepared by ultrasonic assisted impregnation-deposition method. Green Energy Environ. **7**, 1361-1376 (2022).
30. Li, X. et al. Insight into the role of additives in catalytic synthesis of cyclohexylamine from nitrobenzene. Chinese J. Chem. **36**, 1191-1196 (2018).
31. Dinh, L. N. Cairns, G. A. Strickland, R. A. McLean, W. & Maxwell, R. S. Mechanism and kinetic modeling of hydrogenation in the organic getter/palladium catalyst/activated carbon systems. J. Phys. Chem. A **119**, 943-951 (2015).
32. Zhu, W. et al. Fabrication of highly dispersed Pd nanoparticles supported on reduced graphene oxide for solid phase catalytic hydrogenation of 1,4-bis(phenylethynyl) benzene. Intern. J. Hydrogen Energy **45**, 8385-8395 (2020).
33. Zhao, X. et al. Three-dimensional layered porous graphene aerogel hydrogen getters. Intern. J. Hydrogen Energy **34**,15296-15307 (2022).
34. Ortiz-Acosta, D. Moore, T. Safarik, D. J. Hubbard, K. M. & Janicke, M. 3D-printed silicone materials with hydrogen getter capability. Adv. Funct. Mater. **28**, 1707285 (2018).
35. Sangalang, E. A. et al. Hydrogen uptake kinetics of 1,4-bis(phenylethynyl)benzene (DEB) rubberized

coating on silicone foam substrate. ACS Appl. Mater. Interfaces **12**, 3993-4001 (2020).
36. Dong, H. et al. Polymer framework with continuous pores for hydrogen getters: molding and a boost of getter rate. ACS Appl. Polym. Mater. **2**, 3243-3250 (2020).
37. Yue, G. Z.et al. Ultrathin flexible hydrogen getter films with excellent hydrogen uptake and mechanical properties. Intern. J. Hydrogen Energy **48**, 32434-32445 (2023).
